# cIAP1 regulates the EGFR/Snai2 axis in triple-negative breast cancer cells

**DOI:** 10.1038/s41418-018-0100-0

**Published:** 2018-04-19

**Authors:** Maria Teresa Majorini, Giacomo Manenti, Miguel Mano, Loris De Cecco, Annalisa Conti, Patrizia Pinciroli, Enrico Fontanella, Elda Tagliabue, Claudia Chiodoni, Mario Paolo Colombo, Domenico Delia, Daniele Lecis

**Affiliations:** 1 Department of Experimental Oncology and Molecular Medicine, Fondazione IRCCS Istituto Nazionale dei Tumori, Molecular Mechanisms of Cell Cycle Control Unit, Milan, Italy; 2 Department of Predictive & Preventive Medicine, Fondazione IRCCS Istituto Nazionale dei Tumori, Milan, Italy; 3 Functional Genomics and RNA-Based Therapeutics Laboratory, Center for Neuroscience and Cell Biology (CNC), University of Coimbra, Coimbra, 3060-197 Portugal; 4 Functional Genomics and Bioinformatics Core Facility, Department of Experimental Oncology and Molecular Medicine, Fondazione IRCCS Istituto Nazionale dei Tumori, Milan, Italy; 5 Department of Experimental Oncology & Molecular Medicine, Fondazione IRCCS Istituto Nazionale dei Tumori, Molecular Targeting Unit, Milan, Italy; 6 Department of Experimental Oncology and Molecular Medicine, Fondazione IRCCS Istituto Nazionale dei Tumori, Molecular Immunology Unit, Milan, Italy; 7 Present Address: Center for Genomic Science of IIT@SEMM, Fondazione Istituto Italiano di Tecnologia (IIT), Milan, Italy

## Abstract

Inhibitor of apoptosis (IAP) proteins constitute a family of conserved molecules that regulate both apoptosis and receptor signaling. They are often deregulated in cancer cells and represent potential targets for therapy. In our work, we investigated the effect of IAP inhibition in vivo to identify novel downstream genes expressed in an IAP-dependent manner that could contribute to cancer aggressiveness. To this end, immunocompromised mice engrafted subcutaneously with the triple-negative breast cancer MDA-MB231 cell line were treated with SM83, a Smac mimetic that acts as a pan-IAP inhibitor, and tumor nodules were profiled for gene expression. SM83 reduced the expression of Snai2, an epithelial-to-mesenchymal transition factor often associated with increased stem-like properties and metastatic potential especially in breast cancer cells. By testing several breast cancer cell lines, we demonstrated that Snai2 downregulation prevents cell motility and that its expression is promoted by cIAP1. In fact, the chemical or genetic inhibition of cIAP1 blocked epidermal growth factor receptor (EGFR)-dependent activation of the mitogen-activated protein kinase (MAPK) pathway and caused the reduction of Snai2 transcription levels. In a number of breast cancer cell lines, cIAP1 depletion also resulted in a reduction of EGFR protein levels which derived from the decrease of its gene transcription, though, paradoxically, the silencing of cIAP1 promoted EGFR protein stability rather than its degradation. Finally, we provided evidence that IAP inhibition displays an anti-tumor and anti-metastasis effect in vivo. In conclusion, our work indicates that IAP-targeted therapy could contribute to EGFR inhibition and to the reduction of its downstream mediators. This approach could be particularly effective in tumors characterized by high levels of EGFR and Snai2, such as triple-negative breast cancer.

## Introduction

Triple-negative breast cancers (TNBCs) are characterized by the lack of estrogen receptor (ER), progesterone receptor (PR), and HER2 expression, and account for about 15% of all invasive breast cancers [1]. TNBC patients are treated with chemotherapy, usually doxorubicin and taxanes, but do not benefit from endocrine or HER2-directed therapy [1]. Moreover, few intervention opportunities are currently available for the many patients who develop metastatic recurrences. About 80% of TNBCs are defined basal-like according to their gene expression profiles which are reminiscent of breast basal or myoepithelial cells. From an immunophenotypical viewpoint, basal-like cells are characterized by cytokeratin 5/6 and epidermal growth factor receptor (EGFR) positivity [2]. The latter is a key regulator of cell proliferation, survival, and metabolism [3], and its overexpression has been associated with poor clinical outcomes. Nonetheless, anti-EGFR therapy is less effective in breast cancer than in lung, colon, head, and neck cancers [4] and there is therefore the need to fully understand the mechanisms underlying EGFR regulation to design novel targeted strategies.

EGFR exerts its function by modulating many signaling pathways and activating mitogen-activated protein kinases (MAPKs), which in turn promote Snai2 accumulation [5]. Accordingly, this transcription factor is expressed upon EGFR activation [6–10]. Snai2, also known as Slug, first described as an epithelial-to-mesenchymal transition (EMT) regulator capable of inhibiting E-Cadherin expression [11], has also been shown to promote the basal cell program [12, 13], and to play a role in normal mammary gland morphogenesis [14, 15]. Snai2 prevents stem cell differentiation through the functional interaction with other EMT mediators [16]. Moreover, by binding with histone modifying enzymes such as LSD1 [14], it affects the expression of a plethora of genes.

In cancer cells, Snai2 promotes aggressiveness and resistance to therapy [17–19] by favoring cancer cell stem-like [20] and EMT properties [7, 21, 22], especially in breast cancer [23], and it supports metastasis formation by increasing plasticity, cell motility [12] and resistance to detachment-induced cell death. Interestingly, Snai2 knockdown results in reduced invasion and metastasis formation in breast cancer models [24], making Snai2 an attractive target for cancer therapy even though specific inhibitors are not available yet.

Inhibitor of apoptosis proteins (IAPs) constitute a family of molecules which prevent cell death and regulate a number of signaling pathways [25]. IAPs are often deregulated in tumors and have been associated with poor prognosis by increasing cancer cell aggressiveness and resistance to therapy [26]. For this reason, a class of small molecules, called Smac mimetics (SMs), has been designed to target cellular IAP1 (cIAP1), cIAP2, and x-linked IAP (XIAP) [27–29]. These compounds increase the cytotoxic activity of traditional chemotherapy and prevent IAP-mediated activation of several signaling pathways [30]. We have previously demonstrated that SM83, a bivalent SM developed by us, can efficiently deplete cIAP1 and cIAP2 both in vitro and in vivo [29, 31]. By exploiting this molecule, we demonstrate here that cIAP1 is a novel regulator of EGFR expression and signaling. Moreover, we show that cIAP1 inhibition prevents EGFR-dependent expression of Snai2 and therefore the targeting of this IAP represents a new approach to reduce the aggressiveness of tumors characterized by high levels of EGFR and Snai2, such as TNBCs.

## Results

### SM83 treatment affects gene expression of MDA-MB231 primary tumors

**Fig. 1:**
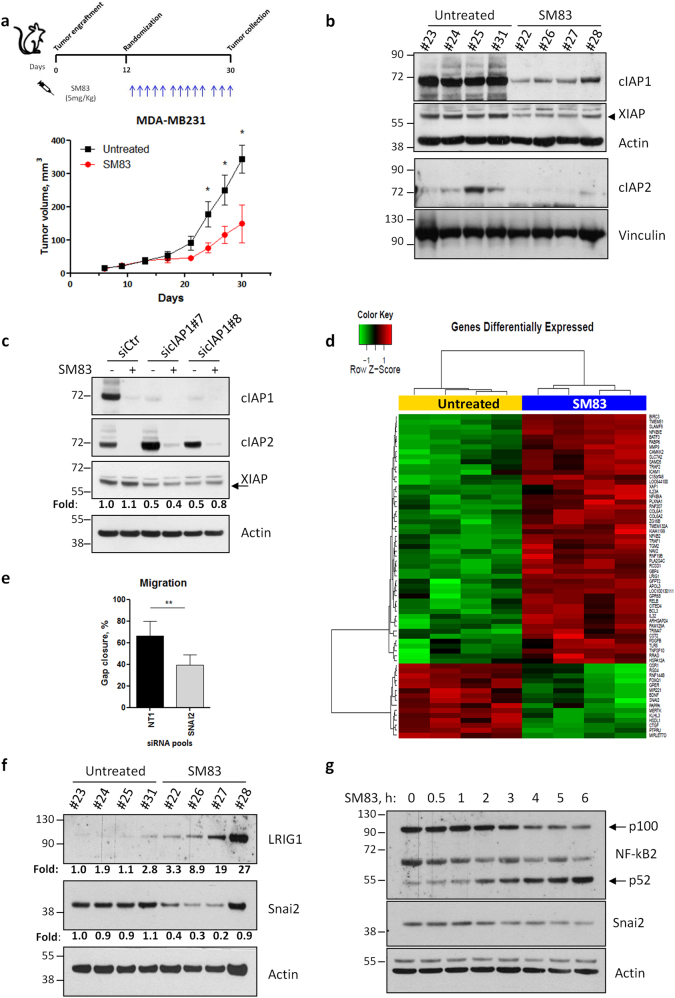
Treatment with SM83 reduces the expression of Snai2 both in vivo and in vitro. **a** NOD/SCID mice engrafted subcutaneously with 5 × 10^6^ MDA-MB231 were treated with intraperitoneal injection of SM83 (5 mg/kg, 5 times/week) or left untreated (4 mice/group) until the end of the experiment. Schedule of the experiment (upper panel) and tumor volumes (bottom panel) measured twice a week (significant differences in days 24, 27, and 30. *P = 0.0476, 0.0391, and 0.0344, respectively. Unpaired two-tailed *t-*test). **b** Six hours after the last injection, mice were killed, nodules collected, and analyzed by western blot to detect the levels of SM83 targets cIAP1, cIAP2, and XIAP. Actin and vinculin are shown as loading controls. **c** MDA-MB231 cells transfected in vitro with two siRNAs targeting cIAP1 were treated or not with 100 nM SM83 for 1 h. Western blots were performed to evaluate the levels of cIAP1, cIAP2, and XIAP 72 h after transfection. Values show the fold levels of XIAP. **d** Differentially expressed genes: heat map showing the 50 genes significantly upregulated and the 15 downregulated by SM83 in MDA-MB231 nodules collected as described in Fig. 1a. **e** Wound-healing experiments performed with MDA-MB231 cells transfected with control (NT1) or Snai2-specific siRNAs (*n* = 4, **P = 0.0033. Unpaired two-tailed *t-*test). The graph shows the percentage of gap closure after 24 h of migration. The complete experiment with the other siRNAs tested is shown in Fig. S2a. **f** The levels of Snai2, downregulated in the GEP shown in Fig. 1d, and LRIG1, upregulated, were evaluated by western blot performed with lysates of MDA-MB231 nodules. Values show the fold levels of Snai2 and LRIG1 normalized to Actin levels. **g** Levels of Snai2 in MDA-MB231 cells treated with 100 nM SM83 in time-course experiments. Cleavage of p100 NF-kB2 into the p52 form was used to verify the expected activation of the non-canonical NF-kB pathway upon SM83 administration

To identify genes that are transcriptionally regulated by IAPs in vivo, we treated NOD/SCID mice bearing subcutaneous triple-negative breast MDA-MB231 tumors with the pan-IAP inhibitor SM83 (Fig. 1a, upper panel) and found out that tumor growth was reduced by about 50% (Fig. 1a, bottom panel). We then tested the efficacy of the treatment at molecular level by evaluating the levels of the known targets of SMs and confirmed that SM83 reduced the levels of cIAP1, cIAP2, and XIAP (Fig. 1b). As SMs are known to trigger the degradative ubiquitination of cIAP1 and cIAP2, but not of XIAP, we investigated the potential mechanism underlying the reduction of XIAP protein levels and concluded that this is likely a consequence of cIAP1 downregulation, rather than being a direct effect of SM83 treatment. In fact, in vitro treatment with SM83 and silencing of cIAP1 were both sufficient to induce XIAP downregulation in MDA-MB231 cells (Fig. 1c).

MDA-MB231 nodules were collected 6 h after the last treatment and profiled for gene expression, which showed 65 genes significantly modified by SM83 treatment, 50 being upregulated, and 15 downregulated (Fig. 1d). As expected, GSEA analysis revealed that SM83 treatment perturbed the expression of genes playing a role in NF-kB regulation or being targets of this pathway (Fig. S1). We focused on the 15 genes downregulated by SM83 and studied the effect of knocking down each of these genes in wound-healing and proliferation experiments, hypothesizing that their reduction could contribute to SM83 anti-tumor activity. By using this approach, we found that Snai2 depletion was the most effective in reducing MDA-MB231 cell motility, with little effect on cell proliferation (Fig. 1e and S2a–b).

We next checked whether SM83 administration affects Snai2 levels also at the protein level and confirmed a marked reduction in primary tumors (Fig. 1f). As a control, we also verified the upregulation of LRIG1 (Fig. 1f), an ubiquitin-ligase known to be upregulated by SM treatment and which we found significantly induced in our gene profiling experiments (Fig. 1d). Finally, the capacity of SM83 treatment to reduce Snai2 was also confirmed in vitro in time-course experiments (Fig. 1g), which showed a gradual downregulation of Snai2, paralleled by the predicted activation of the non-canonical NF-kB pathway.

### Snai2 levels are determined by cIAP1, but not by cIAP2 or XIAP, expression

**Fig. 2:**
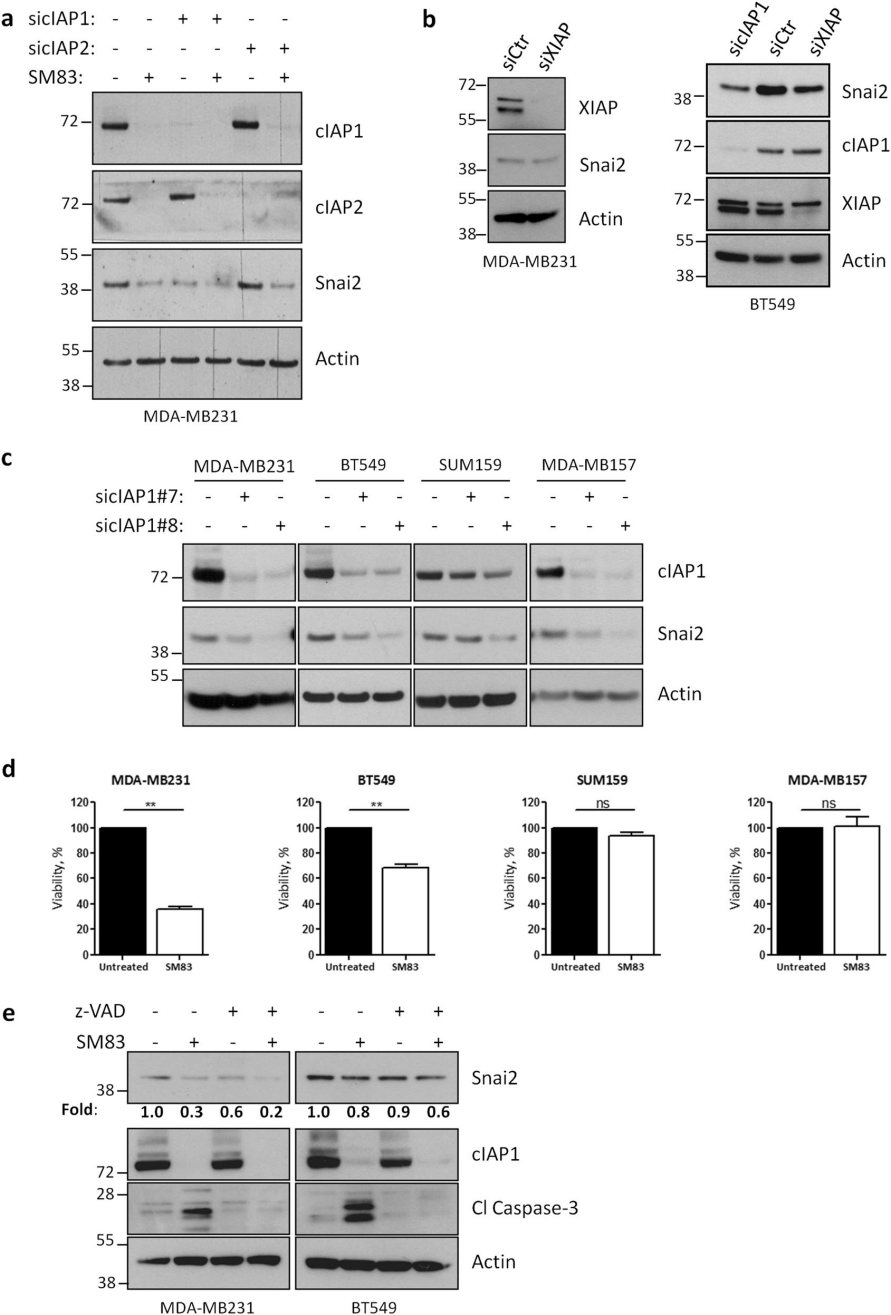
Snai2 expression is promoted by cIAP1, but not cIAP2 or XIAP. **a** Western blot showing the levels of Snai2 in MDA-MB231 cells transfected with siRNAs specific for cIAP1 or cIAP2, and, after 72 h, treated with 100 nM SM83 for further 6 h. **b** MDA-MB231 and BT549 cells were transfected as in Fig. 2a with siRNAs targeting XIAP and cIAP1, and western blot was performed to detect the levels of Snai2. **c** MDA-MB231, BT549, SUM159, and MDA-MB157 cells were transfected with two siRNAs specific for cIAP1 and the levels of Snai2 were detected. **d** The same cell lines employed in Fig. 2c were treated with 100 nM SM83 and viability tested with CellTiter-Glo assay 24 h later (two-tailed paired *t-*test, *n* = 3. MDA-MB231 **P = 0.0011; BT549 **P = 0.0082; SUM159 and MDA-MB157; ns not significant). **e** SM83 (100 nM) was used to treat MDA-MB231 and BT549 cells, pre-treated or not 1 h with 20 µM z-VAD, for 6 h and then cells were analyzed by western blot to detect the total levels of Snai2 and cIAP1, and the cleaved form of caspase-3. Values show the fold levels of Snai2 relative to untreated cells

As SM83 targets different IAPs, we investigated which of the known targets was responsible for SM83-mediated reduction of Snai2 levels. For this reason, cIAP1 and cIAP2 (Fig. 2a) were silenced in MDA-MB231 cells finding that depletion of cIAP1 was sufficient to reduce Snai2 levels, whereas depletion of cIAP2 had no effect. Silencing of XIAP did not affect Snai2 levels in MDA-MB231 cells, while it slightly reduced Snai2 levels in BT549 cells (Fig. 2b), although to a lesser extent compared to cIAP1. Of note, cIAP1 silencing did not affect XIAP levels in BT549 cells in contrast to what found in MDA-MB231 cells (Fig. 1c). The reduction of Snai2 mediated by cIAP1 silencing was further confirmed by using two different siRNAs and employing a number of breast cancer cell lines expressing detectable levels of Snai2 (Fig. 2c). In all the cells lines tested, cIAP1 silencing reduced Snai2 levels, confirming a widely valid regulation of Snai2 by this IAP. Nonetheless, we considered the possibility that Snai2 downregulation could be caused by a toxic effect of IAP targeting. In fact, a small percentage of cancer cell lines are known to be killed by SMs in monotherapy. Therefore, the cell lines transfected in Fig. 2c were treated with 100 nM SM83, finding that MDA-MB231 and BT549 cells were the only ones sensitive to the treatment (Fig. 2d) at this concentration. So, the latter two cell lines were pre-treated with the pan-caspase inhibitor z-VAD, which, however, was not able to prevent the SM83-dependent downregulation of Snai2 (Fig. 2e), suggesting that this reduction is a direct effect of cIAP1 inhibition and not a side effect of toxicity.

### Snai2 expression is stimulated by EGFR in a MAPK-dependent manner

**Fig. 3:**
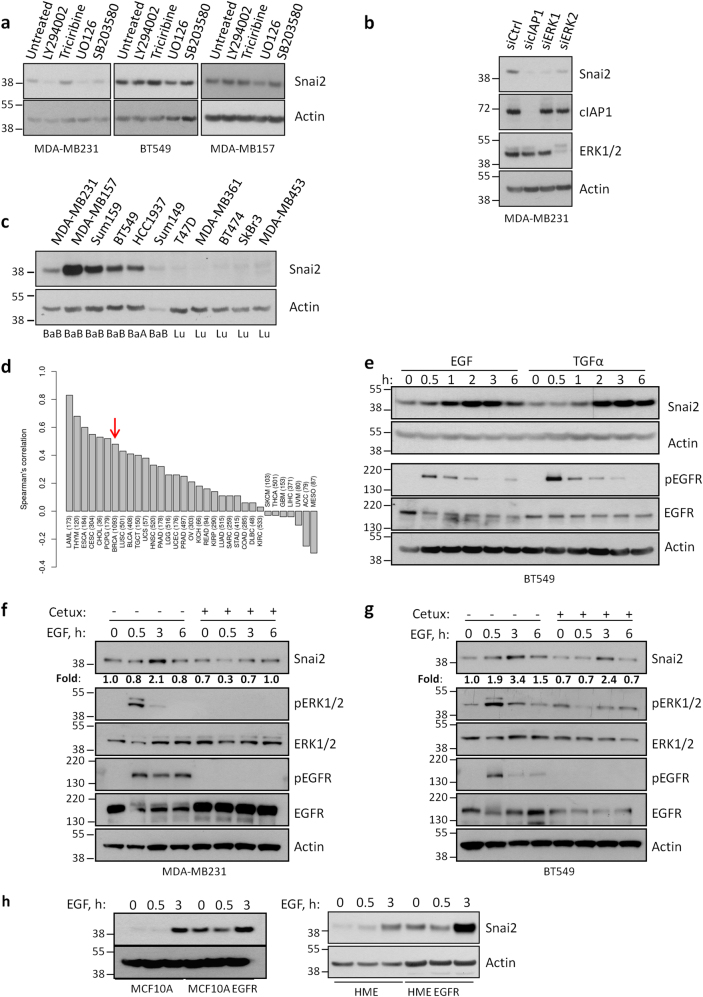
EGFR promotes Snai2 expression in a MAPK-dependent manner. **a** MDA-MB231, BT549, and MDA-MB157 cells were harvested after treatment for 2 h with 10 μM inhibitor of PI3K (LY294002), AKT (Triciribine), MEK (UO126), and p38 (SB203580). Western blot was performed to analyze the levels of Snai2. **b** MDA-MB231 cells were transfected with siRNAs specific for cIAP1, ERK1, and ERK2 to detect the levels of Snai2 72 h after transfection. cIAP1 and ERK1/2 are shown to control the silencing efficiency. **c** A panel of breast cancer cell lines was tested to compare the levels of Snai2. BaA basal “A”, BaB basal “B”, Lu Luminal [55]. **d** For each cancer type available in the TCGA study, Spearman’s correlation between EGFR and SNAI2 was calculated using RNA-Seq data (expressed as log2 counts per million mapped reads). Only primary tumors were considered in the analysis. Red arrow indicates the correlation bar in breast cancers. **e** BT549 cells were serum-starved overnight and then stimulated with 20 ng/ml EGF and TGFα in time-course experiments. Snai2 levels are shown together with total and activated levels of EGFR. MDA-MB231 (**f**) and BT549 (**g**) cells were serum-starved overnight, pre-treated or not with 100 μg/ml cetuximab for 1 h and then stimulated with 20 ng/ml EGF for the indicated time points. Western blot was performed to detect Snai2 levels, total ERK1/2 and EGFR, and their activated levels. Values show the fold levels of Snai2 relative to untreated cells. **h** Human mammary epithelial cell lines, parental and bearing mutated EGFR, were serum-starved and stimulated with 20 ng/ml EGF for the indicated times to evaluate Snai2 levels

As IAPs are apical regulators of several pathways, we questioned which of these pathways were responsible for Snai2 regulation by using specific inhibitors for PI3K, AKT, MEK, and p38. The MEK inhibitor UO126 strongly and reproducibly reduced the expression of Snai2 in all cell lines tested (Fig. 3a) and the same effect was obtained upon ERK1 and ERK2 silencing (Fig. 3b). Importantly, Snai2 is more expressed in basal-like breast cancer cells (Fig. 3c), which are often characterized by increased levels of EGFR, and a correlation exists between EGFR and Snai2 expression in several cancer types, among whom breast cancer (Fig. 3d). Hence, we focused on the EGFR/MAPK pathway and stimulated EGFR with two of its known ligands, EGF and TGFα. As expected, the stimulation increased the expression of Snai2 (Fig. 3e) and this was attenuated by pre-treatment with the EGFR-specific inhibitor cetuximab, which reduced EGFR and ERK1/2 activation, and eventually resulted in limited EGF-dependent stimulation of Snai2 (Fig. 3f, g). Notably, increased levels of Snai2 were also seen in normal mammary epithelial cell lines upon EGFR stimulation or in the presence of constitutive expression of active EGFR mutant (Fig. 3h), suggesting that aberrant activation of EGFR can promote Snai2 expression.

### Depletion of cIAP1 prevents EGFR-dependent activation of ERK1/2 and expression of Snai2

**Fig. 4:**
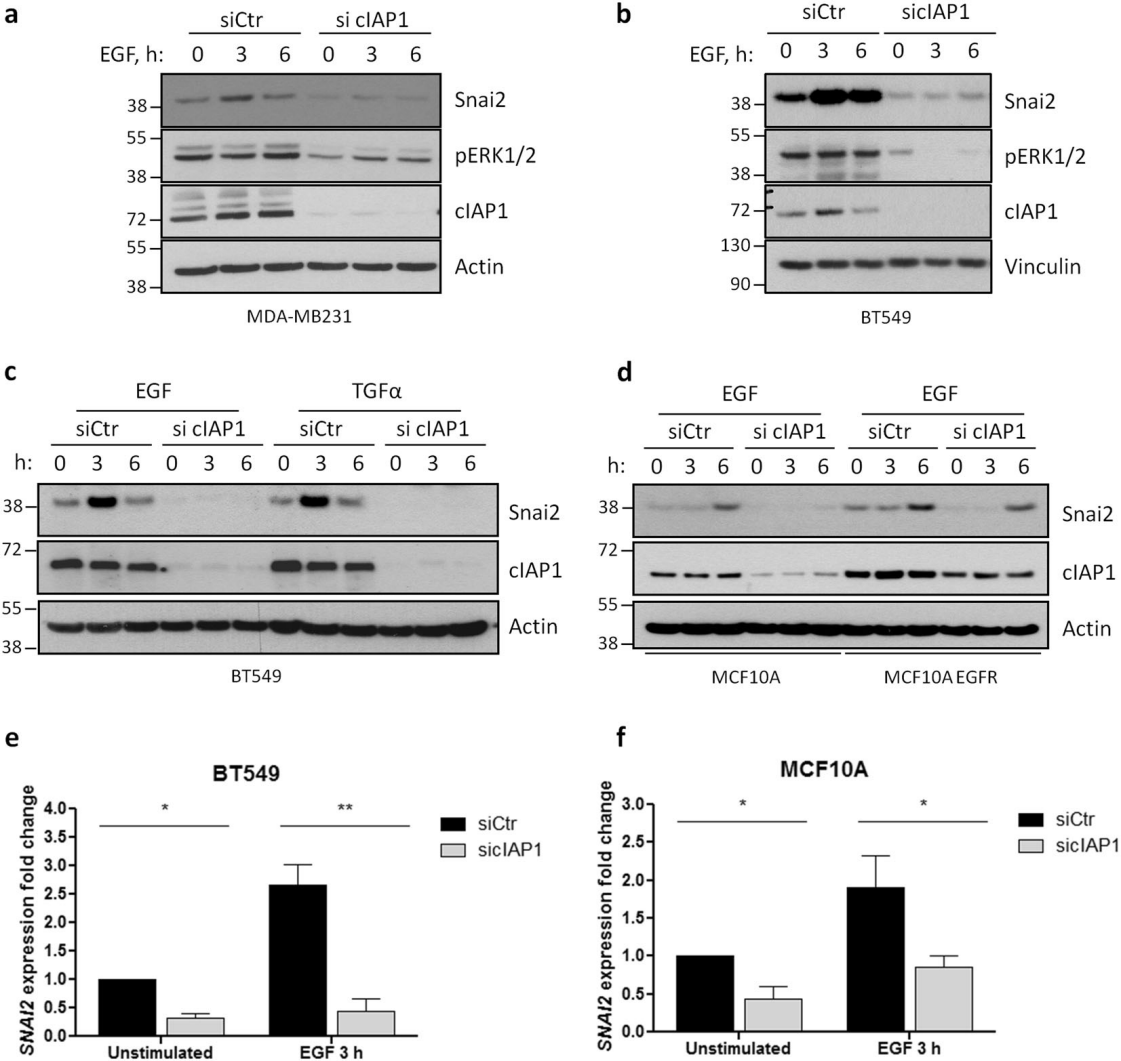
Depletion of cIAP1 hinders EGFR-dependent expression of Snai2. **a** MDA-MB231 and **b** BT549 cells were transfected with control or cIAP1-specific siRNAs and, after 48 h, serum-starved overnight. Then, cells were stimulated for the indicated time points with 50 ng/ml and 20 ng/ml EGF, respectively. Levels of Snai2 and activated ERK1/2 were detected, together with cIAP1, to check the transfection efficiency. **c** BT549 and **d** MCF10A—wild-type or bearing mutated EGFR—cells were transfected as in Fig. 4a and stimulated with the indicated EGFR ligands (20 ng/ml) to evaluate the expression of Snai2 by western blot. **e** BT549 and **f** MCF10A cells were transfected and serum-starved as described before, stimulated 3 h with 20 ng/ml EGF and lysed to extract RNA. Real-time PCR was performed to evaluate Snai2 fold expression relative to GAPDH. BT549: *P = 0.0151, **P = 0.0036; *n* = 3; MCF10A: unstimulated siCtr vs. sicIAP1 *P = 0.0290, EGF 3 h siCtr vs. sicIAP1 *P = 0.0330; *n* = 5; two-tailed paired *t-*test

To elucidate the role of cIAP1 in EGFR regulation and ERK1/2 activation, experiments were performed in MDA-MB231 (Fig. 4a) and BT549 (Fig. 4b) cells silenced for cIAP1 and stimulated with EGF. Depletion of cIAP1 strongly reduced ERK1/2 phosphorylation (Fig. 4a, b), and prevented the accumulation of Snai2 upon EGF or TGFα exposure (Fig. 4c). Snai2 induction was also equally repressed in normal mammary epithelial cells bearing wild-type and mutated EGFR (Fig. 4d). Finally, we checked whether cIAP1 knockdown affected Snai2 accumulation by inhibiting its transcription, as observed in vivo with SM83 treatment, and found that cIAP1 silencing not only reduces the basal levels of Snai2 mRNA, but also prevents its EGFR-dependent increase, both in breast cancer (Fig. 4e) and normal mammary epithelial cells (Fig. 4f).

### Knockdown of cIAP1 reduces EGFR levels

**Fig. 5:**
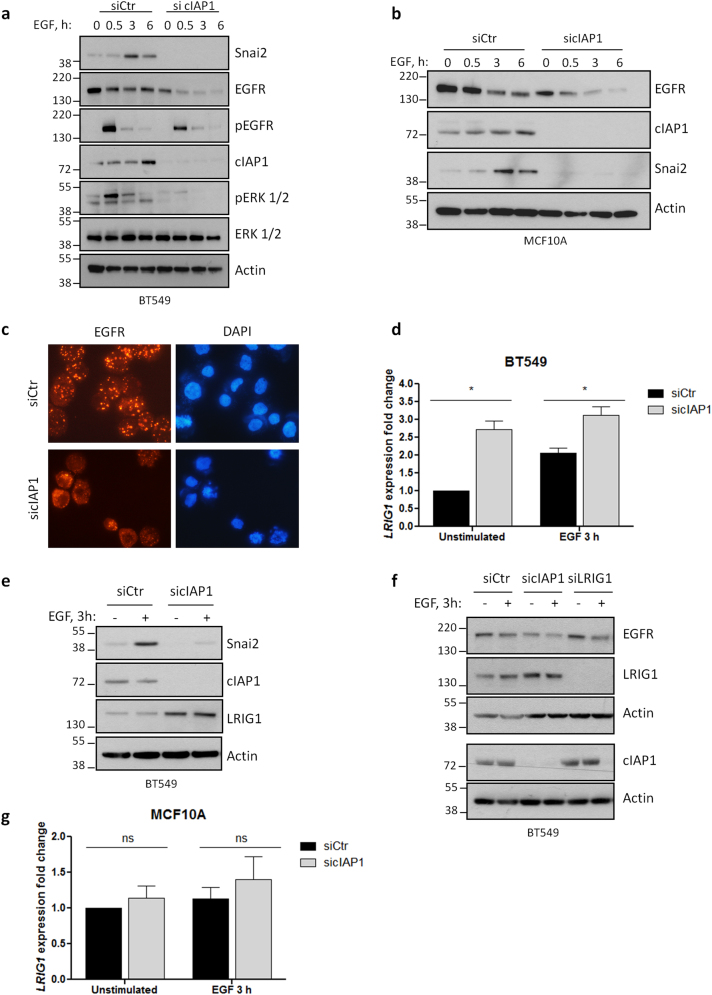
Silencing of cIAP1 reduces EGFR levels. **a** BT549 and **b** MCF10A cells were transfected with control or cIAP1-specific siRNAs before being serum-starved overnight, stimulated with 20 ng/ml EGF and analyzed by western blot to detect the indicate proteins. **c** BT549 cells were transfected with control or cIAP1-specific siRNAs and, after 48 h, cells were serum-starved for 24 h and then stimulated 30 min with 20 ng/ml EGF. Fixed cells were incubated with anti-EGFR antibody and nuclei stained with DAPI. **d** LRIG1 expression levels were evaluated by real-time PCR in BT549 cells serum-starved and stimulated with 20 ng/ml EGF after transfection with control or cIAP1-directed siRNAs. Unstimulated siCtr vs. sicIAP1 *P = 0.0175, EGF 3 h siCtr vs. sicIAP1 *P = 0.0341; *n* = 3; two-tailed paired *t-*test. **e** Levels of Snai2 and LRIG1 in BT549 cells silenced for cIAP1 and stimulated or not with 20 ng/ml EGF. **f** EGFR levels in BT549 cells silenced for cIAP1 and LRIG1, stimulated or not with 20 ng/ml EGF. **g** LRIG1 expression levels were evaluated by real-time PCR in MCF10A cells serum-starved and stimulated with 20 ng/ml EGF after transfection with control or cIAP1-directed siRNAs. ns not significant; *n* = 4; two-tailed paired *t-*test

We then investigated the relationship between cIAP1 and EGFR signaling. In both cancer and normal mammary epithelial cell lines, cIAP1 depletion resulted in a marked drop of EGFR levels (Fig. 5a, b), which further decreased upon stimulation with EGF. Moreover, the ligand-induced EGFR foci detected in control cells were abrogated upon cIAP1 knockdown (Fig. 5c), suggesting an impaired receptor signal transduction. As LRIG1, a known EGFR inhibitor, is upregulated in cells treated with SM83 (Fig. 1d, f), we verified whether it plays a role in cIAP1-dependent regulation of EGFR. In cIAP1-silenced BT549 cells, LRIG1 levels increased both at transcriptional (Fig. 5d) and protein level (Fig. 5e), but, in spite of this, EGFR levels only marginally increased upon LRIG1 depletion (Fig. 5f). Moreover, the transcriptional upregulation of LRIG1 was not evident after cIAP1 knockdown in MCF10A cells (Fig. 5g), which however strongly reduced EGFR levels (Fig. 5b). Taken together, these findings lead us to conclude that LRIG1 upregulation may not be the only factor responsible for EGFR inhibition.

### Depletion of cIAP1 favors EGFR protein stability, but reduces its transcription

**Fig. 6:**
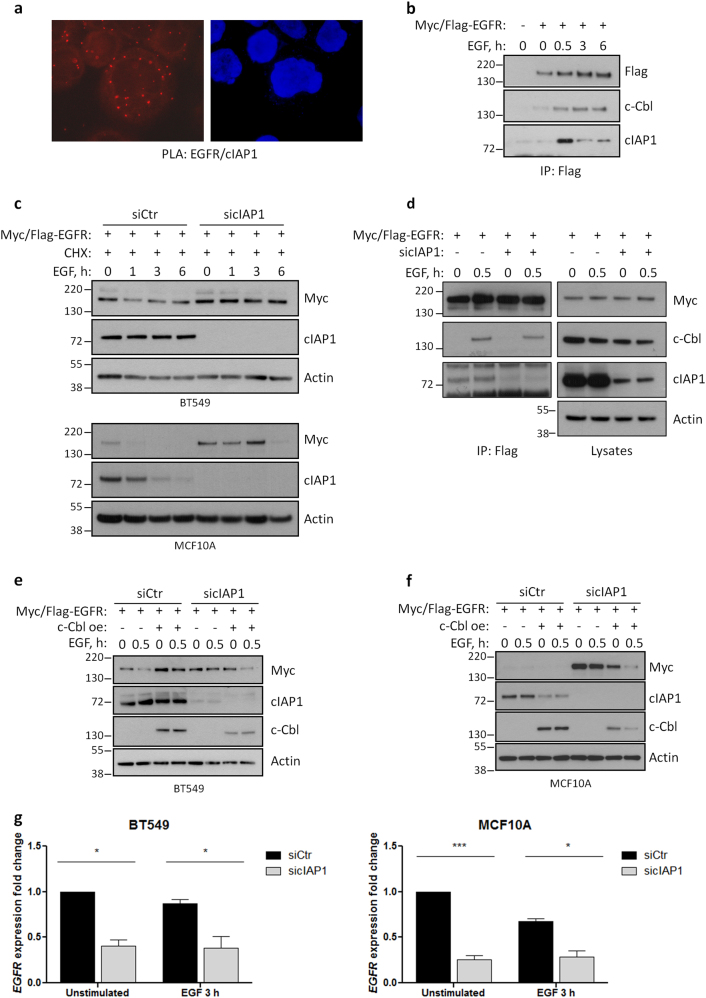
cIAP1 reduces EGFR stability, but promotes its gene transcription. **a** EGFR and cIAP1 interaction was tested in BT549 cells using PLA assay. **b** BT549 cells stably expressing Myc/Flag-tagged EGFR were serum-starved and stimulated with 20 ng/ml EGF for the indicated times. Cells were lysed and EGFR was immunoprecipitated with anti-Flag antibody. Western blot was performed to evaluate the interaction of ectopic EGFR with cIAP1 and c-Cbl. **c** BT549 and MCF10A cells stably expressing Myc/Flag-tagged EGFR were serum-starved, pre-treated with 100 (BT549) or 50 (MCF10A) µg/ml cycloheximide for 30 min and stimulated with 20 ng/ml EGF for the indicated times. Levels of ectopic EGFR were detected with anti-Myc antibody. **d** Ectopic EGFR was immunoprecipitated as described in Fig. 6b from BT549 cells transfected with control and cIAP1-specific siRNAs. Western blot shows total levels of c-Cbl and the amount of c-Cbl interacting with EGFR. **e** BT549 and **f** MCF10A cell stably expressing ectopic Myc/Flag-tagged EGFR were further transduced with lentiviral particles to overexpress c-Cbl or GFP as a control. Cells were silenced for cIAP1, serum-starved, and stimulated with 20 ng/ml EGF as described above, and analyzed by western blot to evaluate the levels of ectopic EGFR by using a Myc-tagged-specific antibody. **g** BT549 (left panel) and MCF10A (right panel) were silenced for cIAP1 and analyzed by real-time PCR to quantify the levels of EGFR expression fold relative to GAPDH. BT549: unstimulated siCtr vs. sicIAP1 *P = 0.0134, EGF 3 h siCtr vs. sicIAP1 *P = 0.0270; *n* = 3. MCF10A: unstimulated siCtr vs. sicIAP1 ***P = 0.0004, EGF 3 h siCtr vs. sicIAP1 **P = 0.0183; *n* = 4; two-tailed paired *t-*test

Having shown that the absence of cIAP1 reduces the levels of EGFR (Fig. 5a, b), we checked whether cIAP1 directly promotes EGFR stability or whether it affects the capacity of c-Cbl, another ubiquitin ligase known to ubiquitinate EGFR, to degrade this receptor. In fact, both cIAP1 and c-Cbl interact with EGFR as show in proximity ligation assay (PLA) and by immunoprecipitation, respectively (Fig. 6a, b). However, cIAP1 depletion surprisingly did not reduce the stability of EGFR, but rather it increased its half-life as demonstrated by blocking protein synthesis with cycloheximide (Fig. 6c). Accordingly, the silencing of cIAP1 slightly reduced the levels and interaction of c-Cbl with EGFR (Fig. 6d), likely contributing to its increased stability. Concordant with this, overexpression of c-Cbl counteracted this effect and reduced the levels of EGFR (Fig. 6e, f) only in cells silenced for cIAP1.

Therefore, we hypothesized that cIAP1 could regulate EGFR transcription levels. Cells were transfected with cIAP1 siRNAs and analyzed by real-time PCR, which confirmed a significant downregulation of EGFR mRNA compared to controls (Fig. 6g). Moreover, as cIAP1 is known to regulate the NF-kB pathway, we investigated whether the latter is responsible for EGFR gene expression and therefore silenced three NF-kB transcription factors. In agreement with our hypothesis, the silencing of p65/RelA resulted in a reduction of EGFR protein levels (Fig. S3).

### The depletion of IAPs prevents EGFR signaling independently of its downregulation

**Fig. 7:**
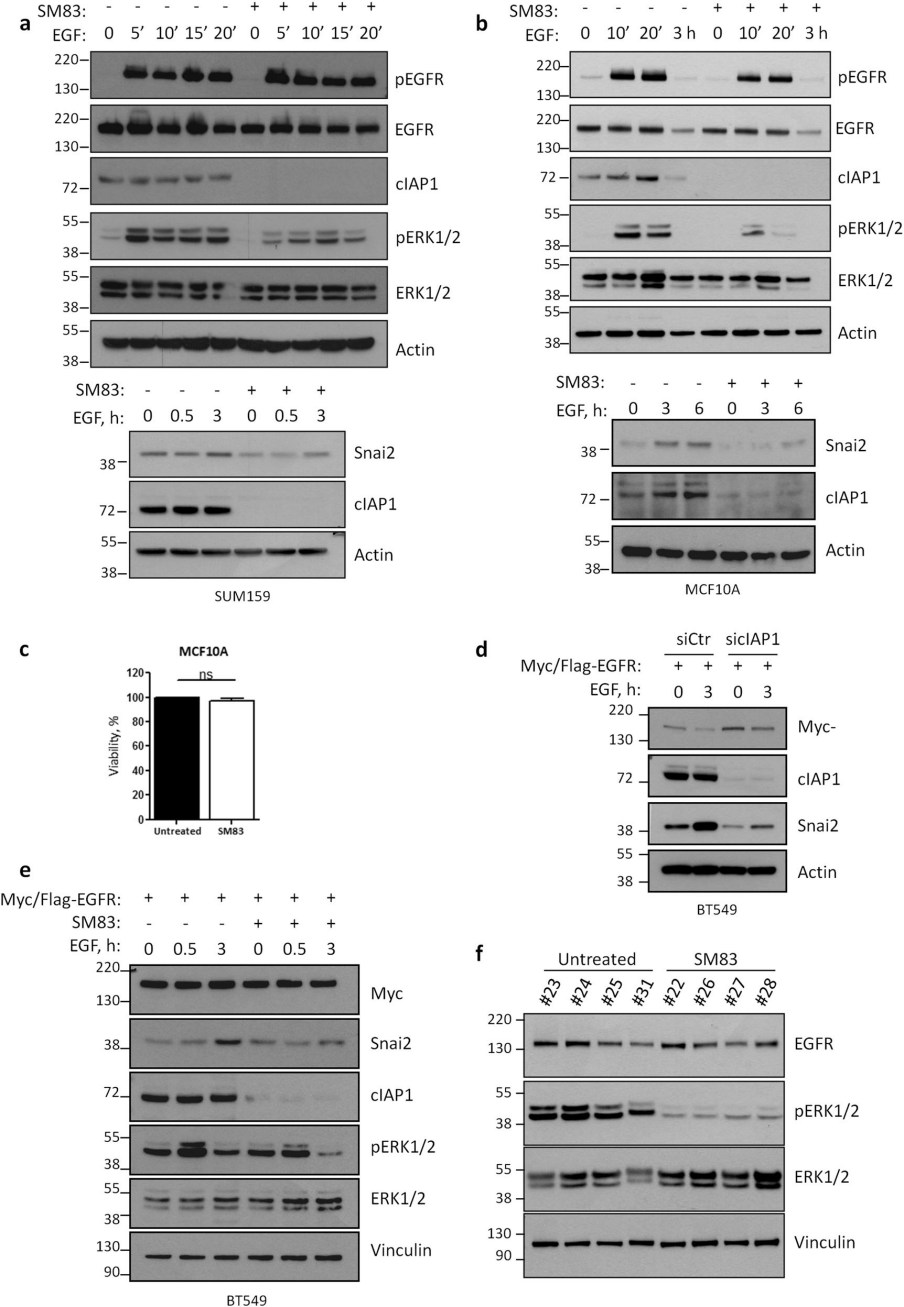
IAP inhibition hinders EGFR signaling independently from the receptor downregulation. **a** SUM159 and **b** MCF10A cells were serum-starved, pre-treated or not with SM83, and stimulated with 20 ng/ml EGF for the indicated times. Western blots were performed to evaluate the total and activated levels of EGFR and ERK1/2, and total levels of Snai2. cIAP1 is shown to control the efficiency of the treatment. **c** MCF10A cell viability was tested by CellTiter-Glo 24 h after treatment with 100 nM SM83. **d** BT549 cells ectopically expressing Myc/Flag-tagged EGFR, silenced with control and cIAP1-specific siRNAs, were serum-starved overnight and then stimulated with 20 ng/ml EGF. Western blot was performed to detect the total levels of ectopic EGFR (Myc), cIAP1, and Snai2. **e** BT549 cells ectopically expressing Myc/Flag-tagged EGFR were serum-starved, pre-treated or not 1 h with SM83, and stimulated with 20 ng/ml EGF for the indicated times. Western blot was performed to detect the total levels of ectopic EGFR (Myc), ERK1/2, cIAP1, and Snai2, and the activated form of ERK1/2. **f** Tumors described in Fig. 1b were analyzed by western blot to detect the activation of ERK1/2, and the total levels of EGFR and ERK1/2

Having observed a reduction of EGFR levels upon cIAP1 silencing, we investigated the effect of IAP targeting in cells treated with SM83. To this end, we focused on SUM159 and MCF10A cells (Fig. 7a, b), which are completely resistant to SM83 treatment in terms of viability (Figs. 2d, 7c). SM83 pre-treatment had no effect on EGFR levels in SUM159 cells (Fig. 7a), and resulted only in a modest, if any, reduction of this receptor in MCF10A cells (Fig. 7b), contrarily to what observed when cIAP1 was silenced for 72 h (Fig. 5b). Nonetheless, the inhibition of IAPs massively prevented the EGF-dependent activation of ERK1/2 and the upregulation of Snai2 in both cell lines (Fig. 7a, b). The effect on ERK1/2 activation was yet minor in MDA-MB157 cells (data not shown), another cell line resistant to SM83 (Fig. 2d). We therefore checked whether the ectopic expression of EGFR could be sufficient to prevent the effect of IAP-targeting in BT549 cells, in which the downregulation of EGFR was evident upon cIAP1 silencing (Fig. 5a). Therefore, BT549 cells stably expressing ectopic EGFR were silenced for cIAP1 or pre-treated with SM83, and then stimulated with EGF. The silencing of cIAP1 prevented the upregulation of Snai2 (Fig. 7d), and the same result was confirmed by SM83-mediated targeting of cIAP1 (Fig. 7e), which also reduced the activation of ERK1/2. Therefore, IAPs, and in particular cIAP1, control the signaling of EGFR even independently of the reduction of its levels. In light of these findings, we analyzed the tumors collected from NOD/SCID mice 6 h after the last SM83 injection (Fig. 1b–f). In our GEP, EGFR was not found among the downregulated genes in vivo, and accordingly EGFR protein levels were not affected by SM83 treatment (Fig. 7f). Nonetheless, ERK1/2 phosphorylation was reduced by the treatment, further supporting the idea that also in vivo ERK1/2 is regulated by IAPs even when EGFR levels are not affected.

### The targeting of IAPs results in anti-tumor and anti-metastasis effect

**Fig. 8:**
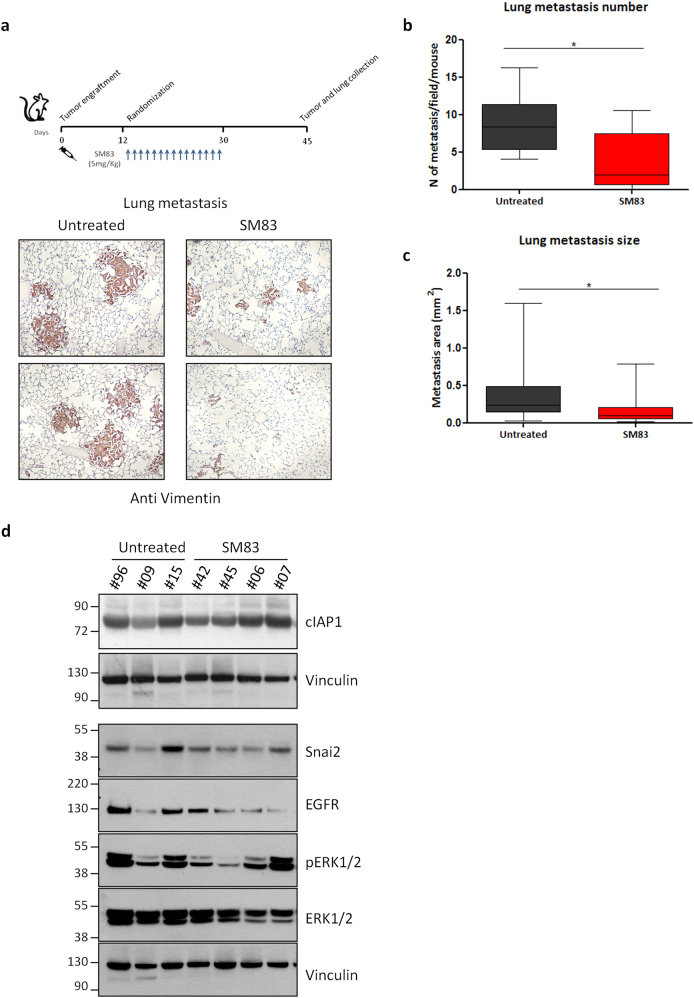
SM83 treatment results in anti-tumor and anti-metastasis effect. **a** Lungs of NOD/SCID mice bearing MDA-MB231 tumors were collected 2 weeks after the last injection with SM83 (upper panel, see Fig. S4a for primary tumor volumes), formalin-fixed and paraffin-embedded, and stained with an anti-human vimentin antibody to detect spontaneous metastasis (bottom panel). **b** Number (untreated *n* = 7, SM83-treated mice *n* = 8; sum of two independent experiments shown in Fig. S4a–b; *P = 0.0238. Unpaired two-tailed *t-*test) and **c** size (35 metastases/group; *P = 0.0107. Unpaired two-tailed *t-*test) of spontaneous MDA-MB231 lung metastases were evaluated. **d** Western blot showing the levels of cIAP1, EGFR, ERK1/2, pERK1/2, and Snai2 in primary tumors at the end of the experiment described in Fig. 8a

Finally, we evaluated the overall effect of IAP-targeting on metastasis formation. In fact, as MDA-MB231 xenografts are known to spontaneously metastasize to lungs, NOD/SCID mice were treated for 3 weeks with SM83 and then lungs were collected after a further 2 weeks (Fig. 7a, upper panel). In accordance to what had been previously observed (Fig. 1a), SM83 treatment delayed the growth of primary tumors (Fig. S4a) and also displayed an anti-metastatic effect (Fig. 8a, bottom panel), resulting in a significant reduction of both number (Fig. 8b) and size (Fig. 8c) of lung metastases. The inhibitory effect of SM83 on the growth of primary tumors and their metastasizing potential was confirmed in additional experiments also employing different administration routes (Fig. S4a–c). Of note, the effect of SM83 was transient and, at the end of the experiments, 2 weeks after the last injection with SM83, cIAP1, Snai2, and phosphorylated ERK1/2 levels were comparable in the primary tumors of either treated or untreated mice (Fig. 8d).

## Discussion

Basal-like breast cancer cells are usually characterized by high expression of the EGFR receptor [2], which is a major regulator of several signaling pathways that sustain cancer cell survival and dissemination. Moreover, EGFR, through the engagement of the MAPK cascade, induces the expression of the EMT-associated factor Snai2 [7, 9]. Here, we show that cIAP1 represents a novel regulator of EGFR by promoting EGFR signaling and expression, while simultaneously favoring its protein degradation. Hence, the targeting of cIAP1 strongly reduces EGFR signaling with consequent inhibition of Snai2 expression, which is a well-known promoter of breast cancer cell aggressiveness (Fig. S5).

Together with their anti-apoptosis activity, IAPs play a pivotal role in the regulation of receptor-mediated signaling [32] being components of TNF-receptor superfamily members [33, 34] and pattern recognition receptors [35]. Accordingly, small molecules designed to inhibit IAPs and trigger cancer cell death also perturb several signaling pathways such as MAPK and NF-kB cascades [30] and modify the expression of a plethora of genes [36, 37]. Accordingly, the in vivo administration of SM83, a SM designed and characterized in our laboratory [29, 31], significantly modulated 65 genes in primary MDA-MB231 nodules. In agreement with other studies, the majority of the perturbed genes are known to be expressed in a NF-kB-dependent manner [36].

Overall, the targeting of IAPs by SM83 resulted in an anti-tumor and anti-metastatic effect. Many different mechanisms could have contributed to this outcome, including direct killing of the cancer cells, effect on the microenvironment [31] and the associated blood vessels [38], as well as perturbation of gene expression. We employed unbiased approaches, which underscored Snai2 as one of the most downregulated genes and showed that its reduction hinders cell motility and, partially, cell proliferation. Snai2 expression was inhibited both in vivo and in vitro by SM83, and, through additional experiments, we identified cIAP1 as the determinant of Snai2 expression. In fact, we provided evidence that the targeting of cIAP1 hinders EGFR signaling by reducing the levels of this receptor and also by preventing the EGFR-mediated activation of ERK1/2. This observation could be clinically relevant as SM treatment might be particularly effective in cancer cells expressing high levels of EGFR, such as breast cancer basal-like cells, in combination with EGFR-targeted therapy [4]. Moreover, IAP-targeting could represent an indirect approach to inhibit Snai2 for which only proof-of-concept molecules [39] or compounds directed against its interactors have been described so far [24, 40]. Inhibition of Snai2 is considered a promising approach to prevent cancer metastasis and resistance to therapy, and there is considerable evidence showing that Snai2-targeting reduces the capability of breast cancer cells to migrate and disseminate to distal organs by reducing their stem-like properties and EMT features [22]. However, further work is still necessary to clarify the function of EMT in metastatisation and the precise role of Snai2 in this process. In fact, recent works have questioned the importance of EMT in metastasis formation [41], also proposing a negligible role of Snai2 in cancer cell metastatic properties [42].

Notably, only a small percentage of cancer cell lines are killed by SMs in monotherapy [43]. Cell death is triggered in a TNF-dependent manner, but cells can acquire resistance to SMs by upregulating LRIG1 [44]. This ubiquitin-ligase is an intestinal stem cell marker and negatively regulates several receptors [45], including Met and EGFR [46]. Therefore, we first investigated the hypothesis that LRIG1 upregulation was responsible for EGFR reduction upon cIAP1 depletion, but paradoxically the protein stability of this receptor was increased by cIAP1 silencing. Therefore, we considered the possibility that other ubiquitin-ligases could regulate EGFR stability and studied the effect of c-Cbl [47] and cIAP1 itself. The latter has recently been shown to regulate the signaling of EGFRvIII variant [48]. Interestingly, cIAP1 depletion increased EGFR stability, supporting the notion that cIAP1 promotes EGFR degradation likely as a consequence of its efficient signaling and internalization. Accordingly, cIAP1 silencing reduced the levels of c-Cbl, which is known to ubiquitinate EGFR and promote its degradation [49, 50]. This effect could contribute to EGFR stabilization and, in support to this hypothesis, c-Cbl ectopic expression counteracted the effect of cIAP1 silencing and restored EGFR degradation.

Despite the increased stability of EGFR, the overall effect of cIAP1 targeting resulted in a strong reduction of the receptor levels in a few cell lines. Our data support the notion that in these cell lines cIAP1 favors EGFR gene expression, and most likely in a NF-kB-dependent manner. Ultimately, cIAP1-depletion-dependent reduction of EGFR could impair MAPK activation and, in turn, reduce the expression of the downstream gene Snai2. Of note, the downregulation of EGFR appeared dispensable for the inhibition of ERK1/2 activation and Snai2 expression upon cIAP1 depletion. In fact, this effect was observed also when depletion of IAPs did not result in a significant reduction of EGFR levels as demonstrated by several lines of evidence. First, when MCF10A and SUM159 cells were exposed to SM83 for a period of time sufficient to deplete cIAP1, but not to reduce EGFR protein levels, ERK1/2 phosphorylation and Snai2 stimulation were still prevented even upon EGF stimulation. In line with this evidence, EGFR ectopic expression was not sufficient to restore ERK1/2 and Snai2 expression in BT549 cells treated with SM83 or silenced for cIAP1. Lastly, MDA-MB231 tumors collected from NOD/SCID mice revealed that SM83 treatment did not affect EGFR levels, but profoundly reduced ERK1/2 activation.

Overall, this work demonstrates that cIAP1 promotes EGFR cascade by sustaining the receptor expression levels and by allowing its downstream signaling, and this effect eventually results in Snai2 expression. Therefore, our work provides new information regarding the mechanisms underlying EGFR regulation and the rationale for combining EGFR inhibitors with IAP-directed small molecules. This approach could be particularly effective in reducing the aggressiveness of cancer cells characterized by the pathological activation of EGFR signaling pathways or high levels of this receptor, as the case of basal-like cells which represent the majority of TNBCs.

## Materials and methods

### Cell culturing and reagents

The human breast adenocarcinoma cell lines MDA-MB231, BT549, MDA-MB157, SkBr3, and HCC1937 cell lines (from American Type Culture Collection) were cultured in vitro with RPMI-1640 medium (Lonza Group, Basel, CH) supplemented with 10% fetal bovine serum (FBS; EuroClone, Milan, IT), 2 mM glutamine, sodium pyruvate, and non-essential amino acids (LONZA). SUM149 and SUM159 (Asterand Bioscience, Detroit, MI), together with the human hTERT-immortalized human mammary epithelial cell line HME—parental and bearing the EGFR delE746A750 mutation (HME EGFR hereafter)—and the human mammary epithelial MCF10A—parental and bearing the EGFR delE746A750 mutation (MCF10A EGFR hereafter)—were cultured as already described [51] in DMEMF-12 (Gibco), supplemented with 10% FBS, 2 mM glutamine, 20 ng/ml EGF (Cat. #GRF-10544, Selleck Chemicals, Munich, D), 10 µg/ml insulin (Sigma-Aldrich), 500 µg/ml hydrocortisone (Sigma-Aldrich). BT474, MDA-MB453, T47D, and MDA-MB361 cell lines were maintained in DMEM (Gibco) supplemented with 10% FBS and 2 mM glutamine. All cells were grown at 37 °C in a humidified incubator with 5% CO_2_. TGFα (#100–16A) was purchased from Peprotech (London, UK), while cycloheximide (CHX, #239764) from Calbiochem. For specific stimulation of EGFR, cells were serum-starved overnight before being stimulated with the indicated concentrations of EGF and TGFα in medium supplemented with 0.1% FBS. HEK293FT (Thermo Fisher Scientific, Milan, IT) for lentiviral production were cultured in DMEM with 10% FBS.

Lipofectamine 2000 (Thermo Fisher Scientific, Waltham, MA, USA) and Lipofectamine RNAiMAX (Thermo Fisher Scientific) reagents were used for plasmid and siRNA transfections, respectively. For silencing experiments, cells were reverse transfected as already described [51]. Cells were treated with the PI3K and AKT (LY294002 and Triciribine, Enzo Life Sciences, Plymouth Meeting, PA), MEK (U0126, Calbiochem, Merck KGaA, Darmstadt, D), and p38 (SB203580, Selleckchem, USA) inhibitors. Cetuximab was provided by the pharmacy of the Istituto Nazionale dei Tumori (Milan, IT). SM83 synthesis has been described elsewhere [29]. Z-VAD(OMe)-FMK was purchased by BIOMOL. Cell viability was measured 24 h after treatment with SM83 by using the CellTiter-Glo Luminescent Cell Viability Assay according to the manufacturer’s protocol (Promega).

### In vivo experiments

Experiments were approved by the Ethics Committee for Animal Experimentation of the Istituto Nazionale Tumori (INT) of Milan according to institutional guidelines and by the Italian Minister of Health (Projects INT_12_2011 and INT_02_2015). Mice were maintained in laminar flow rooms keeping temperature and humidity constant and had free access to food and water. NOD/SCID mice were subcutaneously engrafted with 5 × 10^6^ MDA-MB231 cells and, after 2 weeks, were treated with 5 mg/kg SM83 injected intraperitoneally or intravenously 5 times/week for 3 weeks. Tumor growth was evaluated by biweekly measurements of tumor diameters with a Vernier caliper and tumor volume (TV) was calculated according to the formula: 4/3 × 3.14 × (l/2) x (w/2) x (h/2) where l, w, and h are length, width, and height, respectively. A good correlation was found between TVs predicted with this formula and actual tumor weight measured post-mortem (Fig. S4d). Animals were killed 6 h after the last injection and tumors collected for biochemical and gene expression analysis. In metastasis studies, NOD/SCID mice were killed 2 weeks after the last SM83 administration and, together with subcutaneous nodules, lungs were collected and formalin-fixed/paraffin-embedded for IHC detection of metastasis with anti-human vimentin antibody (M0725, DAKO).

### Gene expression profiling

GEP was performed by the Functional Genomics and Bioinformatic Core Facility Illumina of INT by using the Human HT-12_V4_0_R2 platform (tot. 47323 probes). Analysis was performed through R 2.15.2, with “lumi” and “limma” packages from Bioconductor. Only probes with Absolute Fold Change >1.5 and FDR <0.05 were considered significant. Expression profiles are deposited in the Gene Expression Omnibus Repository, GSE98691 accession number.

### Gene silencing and overexpression

FlexiTube siRNAs specific for cIAP1 (Hs_BIRC2_7 and 8), cIAP2 (Hs_BIRC2_8 and 9), ERK1 (Hs_MAPK3_3 and 7), ERK2 (Hs_MAPK1_ 9 and 10), NFk-B2 (Hs_NFKB2_1), NF-kB1 (Hs_NFKB1_3), RELA (Hs_RELA_5), LRIG1 (Hs_LRIG1_5 and 6) were purchased from Qiagen (Valencia, CA, USA). The control siRNA (siCtr) was synthesized by Eurofins Genomics (sequence 5′-CGUACGCGGAAUACUUCGATT-3′), while siGENOME SMARTpool for XIAP (M-004098-01), Snai2 (M-017386-00) and its control, siGENOME Non-Targeting siRNA Pool #1 (NT1; D-001206-13), were from Dharmacon (GE Healthcare, Lafayette, CO, USA).

To ectopically express EGFR and c-Cbl, cells were transduced with lentiviral particles prepared by HEK293FT packaging cells as already described [52]. The plasmid for human EGFR (Myc/Flag-tagged) ectopic expression was purchased by Origene Company (#RC217384L1; Rockville, MD, USA), while the c-Cbl-expressing vector (RRL-CMV-CBL) was kindly provided by Prof. Pier Paolo Di Fiore. To overexpress cIAP1, cells were transfected with pcdna3.1 plasmids [53] kindly provided by Prof. Jon Ashwell (Addgene plasmid # 8311).

### Western blot and immunoprecipitations

For western blot analysis, cells were harvested, washed twice with ice-cold PBS, and boiled for 10 min in lysis buffer (125 mM Tris HCl, pH 6.8, 5% sodium dodecyl sulfate, SDS) and then added with protease inhibitors. Samples were sonicated, clarified at room temperature by centrifugation for 15 min at 11,000 rpm and then quantified with microBCA (Euroclone). Cleared supernatants were separated by SDS-PAGE on precast 4–12% Bis-Tris NuPAGE gels (Thermo Fisher Scientific) and proteins were transferred to PVDF membranes (Merck Millipore, Darmstadt, Germany) using the XCell II blot module (Thermo Fisher Scientific). Membranes were then saturated for 30 min in Tris-buffered saline containing 4% BSA and incubated overnight with the anti-human primary antibodies recognizing cIAP1 #ab108361, total EGFR #ab30 (Abcam); Snai2 #9585, LRIG1 #12752, NF-kB2 #4882, NF-kB1 #3035, Cleaved Caspase3 #9501, p65/RelA #3034, pEGFR Tyr1068 #2236, c-Cbl #2747, Myc-Tag #2278 (Cell Signaling Technology, Danvers, MA, USA); phospho-ERK1/2 #M8159, ERK1/2 #M5670, Actin #A1978 and Vinculin #V9131 (Sigma-Aldrich), cIAP2 #552783 and XIAP #610763 (BD Biosciences). After incubation with the proper secondary antibody (anti-rabbit and anti-mouse IgG were purchased from GE Healthcare, UK, and anti-goat IgG from Thermo Fisher Scientific; all secondary antibodies were diluted 1:2000), proteins were detected by chemiluminescence (EuroClone).

For FLAG-tagged EGFR immunoprecipitations, BT549 cells were lysed in E1A lysis buffer (ELB) buffer (150 mM NaCl, 50 mM Hepes, pH 7.5, 5 mM EDTA, 0.5% NP40) at 4 °C for 30 min and 1 mg of total protein extract was incubated at 4 °C for 3 h with Flag-M2 Affinity Gel (#A2220, Sigma-Aldrich) previously washed with ELB buffer three times. The bound polypeptides were analyzed by SDS-PAGE and immunoblotting.

### Migration assay

MDA-MB231 cells were reverse transfected in Ibidi inserts (4 × 10^4^ cells/chamber) and motility tested 72 h later in wound-healing experiments performed as already described [54]. Image acquisition and data analysis were carried out with a Cell-IQ instrument (CM-Technologies) and the integrated software.

### RNA extraction and real-time PCR

For real-time PCR, total RNA was extracted from cells with the miRNeasy mini columns (Qiagen) and 1 µg RNA was reverse-transcribed using the Transcriptor First Strand c-DNA Synthesis kit (Roche, Indianapolis, IN, USA). Probes for human SNAI2 (Hs_00950344_m1), LRIG1 (Hs_00394267_m1), EGFR (Hs_01076090_m1), and GAPDH (Hs_02758991_g1) were purchased from Applied Biosystems (Foster City, USA). Real-time PCR was carried out in triplicate using TaqMan Master Mix (Applied Biosystems) and samples were analyzed through the ViiA 7 Real-Time PCR System (Applied Biosystems). All transcript levels were normalized to that of GAPDH.

### Immunofluorescence and PLA

Cells were fixed for 10 min with 4% paraformaldehyde, permeabilized for 10 min at room temperature with 0.2% Triton X-100 and blocked in PBS, 3% BSA, 0.1% Tween-20. Cover slips were then incubated overnight at 4 °C in a humidified chamber with antibodies directed to EGFR (mouse, dilution 1:200, abcam). Fluorescence images were acquired using a fluorescence microscopy and digital image acquisition on a Nikon Eclipse E1000 equipped with a DSU3 CCD camera. For PLA assay, cover slips were stained with anti EGFR (mouse, dilution 1:200, abcam) and/or cIAP1 (rabbit, dilution 1:2000, abcam). After the overnight incubation with primary antibodies, cells were stained with the PLA probes diluted 1:5 in antibody diluent (Sigma-Aldrich) in a humidified chamber for 1 h at 37 °C. Subsequent hybridization, ligation, amplification, and detection were performed as described in the manufacturer’s protocol (Duolink In Situ PLA; Sigma-Aldrich).

### Graphs, statistical analysis, and image analysis

Graphs and statistical analysis was performed using GraphPad Prism 5.02. A value of *P* < 0.05 was considered statistically significant. Western blot densitometric analysis was performed with ImageQuant 5.2. Graphs showing the average of different experiments are shown in Supplementary Fig. S6. GEP analyses were performed as previously described.

## Electronic supplementary material

Figure Supplementary S1(TIF 1428 kb)

Figure Supplementary S2(TIF 1723 kb)

Figure Supplementary S3(TIF 993 kb)

Figure Supplementary S4(TIF 2116 kb)

Figure Supplementary S5(TIF 1889 kb)

Figure Supplementary S6(TIF 1361 kb)

Supplementary figure legends(DOCX 14 kb)

## References

[1] Foulkes WD, Smith IE, Reis-Filho JS (2010). Triple-negative breast cancer. N Engl J Med.

[2] Cheang MC, Voduc D, Bajdik C, Leung S, McKinney S, Chia SK (2008). Basal-like breast cancer defined by five biomarkers has superior prognostic value than triple-negative phenotype. Clin Cancer Res.

[3] Lim SO, Li CW, Xia W, Lee HH, Chang SS, Shen J (2016). EGFR signaling enhances aerobic glycolysis in triple-negative breast cancer cells to promote tumor growth and immune escape. Cancer Res.

[4] Nakai K, Hung MC, Yamaguchi H (2016). A perspective on anti-EGFR therapies targeting triple-negative breast cancer. Am J Cancer Res.

[5] Virtakoivu R, Mai A, Mattila E, De Franceschi N, Imanishi SY, Corthals G (2015). Vimentin-ERK signaling uncouples Slug gene regulatory function. Cancer Res.

[6] Sheu JJ, Lee CC, Hua CH, Li CI, Lai MT, Lee SC (2014). LRIG1 modulates aggressiveness of head and neck cancers by regulating EGFR-MAPK-SPHK1 signaling and extracellular matrix remodeling. Oncogene.

[7] Joannes A, Grelet S, Duca L, Gilles C, Kileztky C, Dalstein V (2014). Fhit regulates EMT targets through an EGFR/Src/ERK/Slug signaling axis in human bronchial cells. Mol Cancer Res.

[8] Zhang Z, Yang M, Chen R, Su W, Li P, Chen S (2014). IBP regulates epithelial-to-mesenchymal transition and the motility of breast cancer cells via Rac1, RhoA and Cdc42 signaling pathways. Oncogene.

[9] Kusewitt DF, Choi C, Newkirk KM, Leroy P, Li Y, Chavez MG (2009). Slug/Snai2 is a downstream mediator of epidermal growth factor receptor-stimulated reepithelialization. J Invest Dermatol.

[10] Arnoux V, Nassour M, L’Helgoualc’h A, Hipskind RA, Savagner P (2008). Erk5 controls Slug expression and keratinocyte activation during wound healing. Mol Biol Cell.

[11] Hajra KM, Chen DY, Fearon ER (2002). The SLUG zinc-finger protein represses E-cadherin in breast cancer. Cancer Res.

[12] Phillips S, Kuperwasser C (2014). SLUG: critical regulator of epithelial cell identity in breast development and cancer. Cell Adh Migr.

[13] Storci G, Sansone P, Trere D, Tavolari S, Taffurelli M, Ceccarelli C (2008). The basal-like breast carcinoma phenotype is regulated by SLUG gene expression. J Pathol.

[14] Phillips S, Prat A, Sedic M, Proia T, Wronski A, Mazumdar S (2014). Cell-state transitions regulated by SLUG are critical for tissue regeneration and tumor initiation. Stem Cell Rep.

[15] Nassour M, Idoux-Gillet Y, Selmi A, Come C, Faraldo ML, Deugnier MA (2012). Slug controls stem/progenitor cell growth dynamics during mammary gland morphogenesis. PLoS ONE.

[16] Puisieux A, Brabletz T, Caramel J (2014). Oncogenic roles of EMT-inducing transcription factors. Nat Cell Biol.

[17] Jiang T, Gao G, Fan G, Li M, Zhou C (2015). FGFR1 amplification in lung squamous cell carcinoma: a systematic review with meta-analysis. Lung Cancer.

[18] Chang TH, Tsai MF, Su KY, Wu SG, Huang CP, Yu SL (2011). Slug confers resistance to the epidermal growth factor receptor tyrosine kinase inhibitor. Am J Respir Crit Care Med.

[19] Findlay VJ, Wang C, Nogueira LM, Hurst K, Quirk D, Ethier SP (2014). SNAI2 modulates colorectal cancer 5-fluorouracil sensitivity through miR145 repression. Mol Cancer Ther.

[20] Luanpitpong S, Li J, Manke A, Brundage K, Ellis E, McLaughlin SL (2016). SLUG is required for SOX9 stabilization and functions to promote cancer stem cells and metastasis in human lung carcinoma. Oncogene.

[21] Claperon A, Guedj N, Mergey M, Vignjevic D, Desbois-Mouthon C, Boissan M (2012). Loss of EBP50 stimulates EGFR activity to induce EMT phenotypic features in biliary cancer cells. Oncogene.

[22] Kao SH, Wang WL, Chen CY, Chang YL, Wu YY, Wang YT (2014). GSK3beta controls epithelial-mesenchymal transition and tumor metastasis by CHIP-mediated degradation of Slug. Oncogene.

[23] Bhat-Nakshatri P, Appaiah H, Ballas C, Pick-Franke P, Goulet R, Jr., Badve S, et al. SLUG/SNAI2 and tumor necrosis factor generate breast cells with CD44+/CD24- phenotype. BMC Cancer. 2010;10:411. 10.1186/1471-2407-10-411 PMC3087321 20691079

[24] Ferrari-Amorotti G, Chiodoni C, Shen F, Cattelani S, Soliera AR, Manzotti G (2014). Suppression of invasion and metastasis of triple-negative breast cancer lines by pharmacological or genetic inhibition of slug activity. Neoplasia.

[25] Gyrd-Hansen M, Meier P (2010). IAPs: from caspase inhibitors to modulators of NF-kappaB, inflammation and cancer. Nat Rev Cancer.

[26] Fulda S (2014). Molecular pathways: targeting inhibitor of apoptosis proteins in cancer–from molecular mechanism to therapeutic application. Clin Cancer Res.

[27] Varfolomeev E, Blankenship JW, Wayson SM, Fedorova AV, Kayagaki N, Garg P (2007). IAP antagonists induce autoubiquitination of c-IAPs, NF-κB activation, and TNFα-dependent apoptosis. Cell.

[28] Vince JE, Wong WW, Khan N, Feltham R, Chau D, Ahmed AU (2007). IAP antagonists target cIAP1 to induce TNFα-dependent apoptosis. Cell.

[29] Lecis D, Mastrangelo E, Belvisi L, Bolognesi M, Civera M, Cossu F (2012). Dimeric Smac mimetics/IAP inhibitors as in vivo-active pro-apoptotic agents. Part II: structural and biological characterization. Bioorg Med Chem.

[30] Varfolomeev E, Goncharov T, Maecker H, Zobel K, Komuves LG, Deshayes K (2012). Cellular inhibitors of apoptosis are global regulators of NF-kappaB and MAPK activation by members of the TNF family of receptors. Sci Signal.

[31] Lecis D, De Cesare M, Perego P, Conti A, Corna E, Drago C (2013). Smac mimetics induce inflammation and necrotic tumour cell death by modulating macrophage activity. Cell Death Dis.

[32] Estornes Y, Bertrand MJ (2015). IAPs, regulators of innate immunity and inflammation. Semin Cell Dev Biol.

[33] Feoktistova M, Geserick P, Kellert B, Dimitrova DP, Langlais C, Hupe M (2011). cIAPs block ripoptosome formation, a RIP1/Caspase-8 containing intracellular cell death complex differentially regulated by cFLIP isoforms. Mol Cell.

[34] Emmerich CH, Schmukle AC, Haas TL, Gerlach B, Cordier SM, Rieser E (2011). The linear ubiquitin chain assembly complex forms part of the TNF-R1 signalling complex and is required for effective TNF-induced gene induction and prevents TNF-induced apoptosis. Adv Exp Med Biol.

[35] Sharma S, Kaufmann T, Biswas S (2017). Impact of inhibitor of apoptosis proteins on immune modulation and inflammation. Immunol Cell Biol.

[36] Chesi M, Mirza NN, Garbitt VM, Sharik ME, Dueck AC, Asmann YW (2016). IAP antagonists induce anti-tumor immunity in multiple myeloma. Nat Med.

[37] Beug ST, Beauregard CE, Healy C, Sanda T, St-Jean M, Chabot J, et al. Smac mimetics synergize with immune checkpoint inhibitors to promote tumour immunity against glioblastoma. Nat Commun. 2017; 8. 10.1038/ncomms14278. 10.1038/ncomms14278 PMC5330852 28198370

[38] Witt A, Seeger JM, Coutelle O, Zigrino P, Broxtermann P, Andree M (2015). IAP antagonization promotes inflammatory destruction of vascular endothelium. EMBO Rep.

[39] Harney AS, Lee J, Manus LM, Wang P, Ballweg DM, LaBonne C (2009). Targeted inhibition of Snail family zinc finger transcription factors by oligonucleotide-Co(III) Schiff base conjugate. Proc Natl Acad Sci USA.

[40] Ferrari-Amorotti G, Fragliasso V, Esteki R, Prudente Z, Soliera AR, Cattelani S (2013). Inhibiting interactions of lysine demethylase LSD1 with snail/slug blocks cancer cell invasion. Cancer Res.

[41] Fischer KR, Durrans A, Lee S, Sheng J, Li F, Wong ST (2015). Epithelial-to-mesenchymal transition is not required for lung metastasis but contributes to chemoresistance. Nature.

[42] Ye X, Tam WL, Shibue T, Kaygusuz Y, Reinhardt F, Ng Eaton E (2015). Distinct EMT programs control normal mammary stem cells and tumour-initiating cells. Nature.

[43] Petersen SL, Wang L, Yalcin-Chin A, Li L, Peyton M, Minna J (2007). Autocrine TNFα signaling renders human cancer cells susceptible to Smac-mimetic-induced apoptosis. Cancer Cell.

[44] Bai L, McEachern D, Yang CY, Lu J, Sun H, Wang S (2012). LRIG1 modulates cancer cell sensitivity to Smac mimetics by regulating TNFalpha expression and receptor tyrosine kinase signaling. Cancer Res.

[45] Wang Y, Poulin EJ, Coffey RJ (2013). LRIG1 is a triple threat: ERBB negative regulator, intestinal stem cell marker and tumour suppressor. Br J Cancer.

[46] Yokdang N, Hatakeyama J, Wald JH, Simion C, Tellez JD, Chang DZ (2016). LRIG1 opposes epithelial-to-mesenchymal transition and inhibits invasion of basal-like breast cancer cells. Oncogene.

[47] Sigismund S, Algisi V, Nappo G, Conte A, Pascolutti R, Cuomo A (2013). Threshold-controlled ubiquitination of the EGFR directs receptor fate. EMBO J.

[48] Puliyappadamba VT, Chakraborty S, Chauncey SS, Li L, Hatanpaa KJ, Mickey B (2013). Opposing effect of EGFRWT on EGFRvIII-mediated NF-kappaB activation with RIP1 as a cell death switch. Cell Rep.

[49] Capuani F, Conte A, Argenzio E, Marchetti L, Priami C, Polo S (2015). Quantitative analysis reveals how EGFR activation and downregulation are coupled in normal but not in cancer cells. Nat Commun.

[50] Fortian A, Dionne LK, Hong SH, Kim W, Gygi SP, Watkins SC (2015). Endocytosis of ubiquitylation-deficient EGFR mutants via clathrin-coated pits is mediated by ubiquitylation. Traffic.

[51] Conti A, Majorini MT, Fontanella E, Bardelli A, Giacca M, Delia D (2017). Lemur tyrosine kinase 2 (LMTK2) is a determinant of cell sensitivity to apoptosis by regulating the levels of the BCL2 family members. Cancer Lett.

[52] Conti A, Majorini MT, Elliott R, Ashworth A, Lord CJ, Cancelliere C (2015). Oncogenic KRAS sensitizes premalignant, but not malignant cells, to Noxa-dependent apoptosis through the activation of the MEK/ERK pathway. Oncotarget.

[53] Li X, Yang Y, Ashwell JD (2002). TNF-RII and c-IAP1 mediate ubiquitination and degradation of TRAF2. Nature.

[54] Colombo C, Minna E, Rizzetti MG, Romeo P, Lecis D, Persani L, et al. The modifier role of RET-G691S polymorphism in hereditary medullary thyroid carcinoma: functional characterization and expression/penetrance studies. Orphanet J Rare Dis. 2015;10:25. 10.1186/s13023-015-0231-z PMC4373282 25887804

[55] Neve RM, Chin K, Fridlyand J, Yeh J, Baehner FL, Fevr T (2006). A collection of breast cancer cell lines for the study of functionally distinct cancer subtypes. Cancer Cell.

