# Supplementary figures and images for: cIAP1 regulates the EGFR/Snai2 axis in triple-negative breast cancer cells
Source: Cell Death Differ. 2018 Apr 19;25(12):2147–64. doi: 10.1038/s41418-018-0100-0 (PMC6262016; doi:10.1038/s41418-018-0100-0)

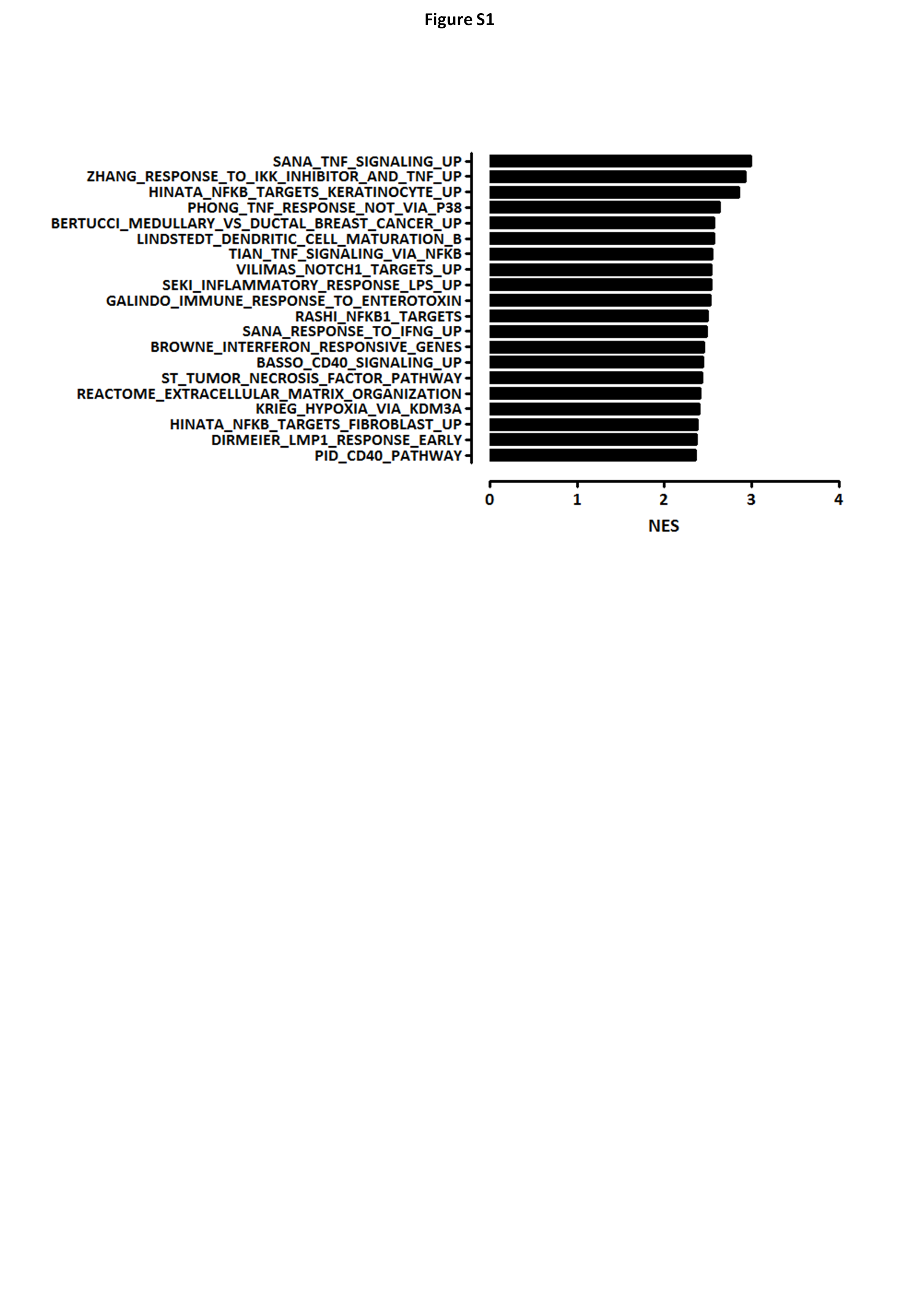

Supplement: Supplementary file 1 — Figure Supplementary S1(TIF 1428 kb) [file 41418_2018_100_MOESM1_ESM.tif]

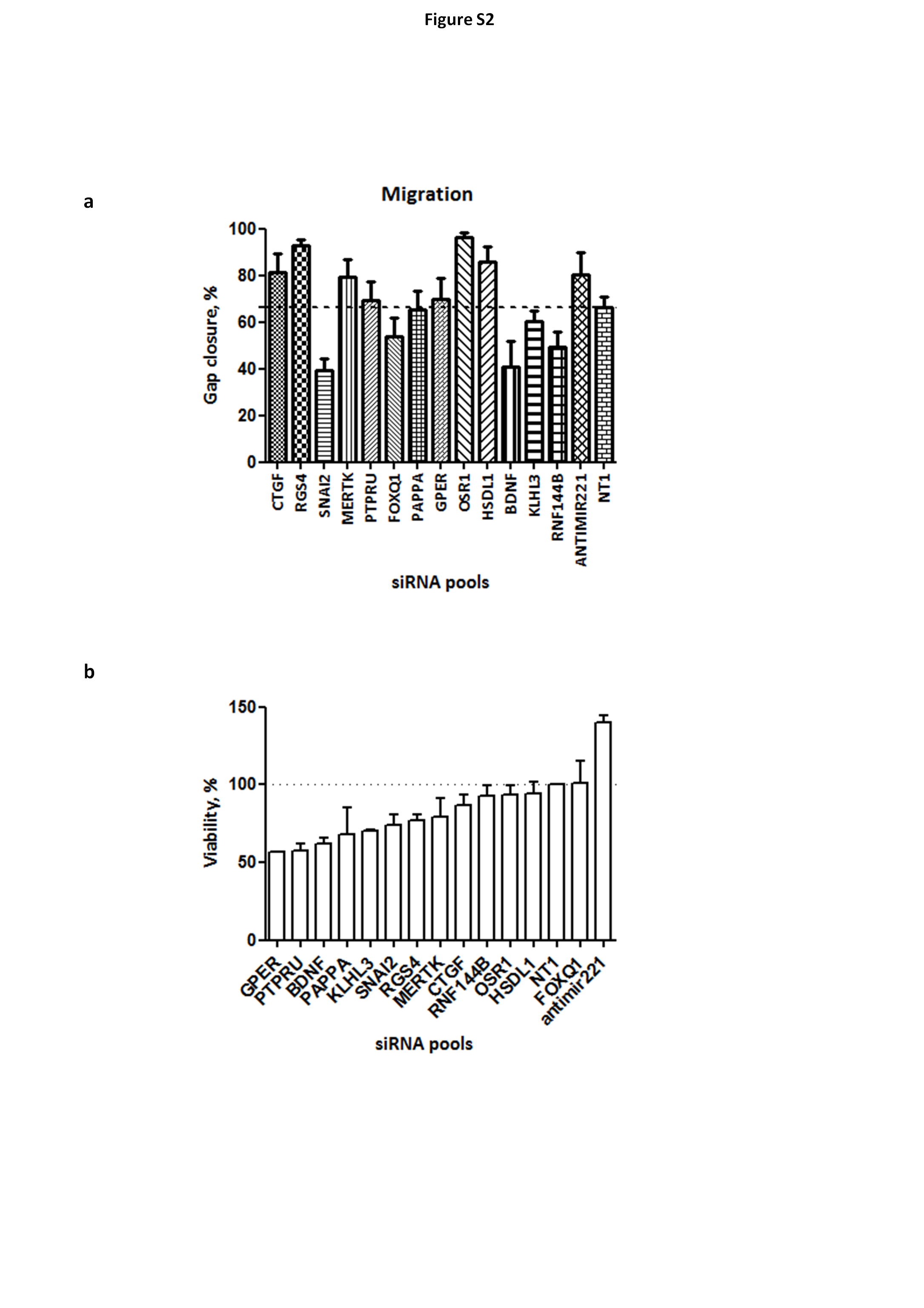

Supplement: Supplementary file 2 — Figure Supplementary S2(TIF 1723 kb) [file 41418_2018_100_MOESM2_ESM.tif]

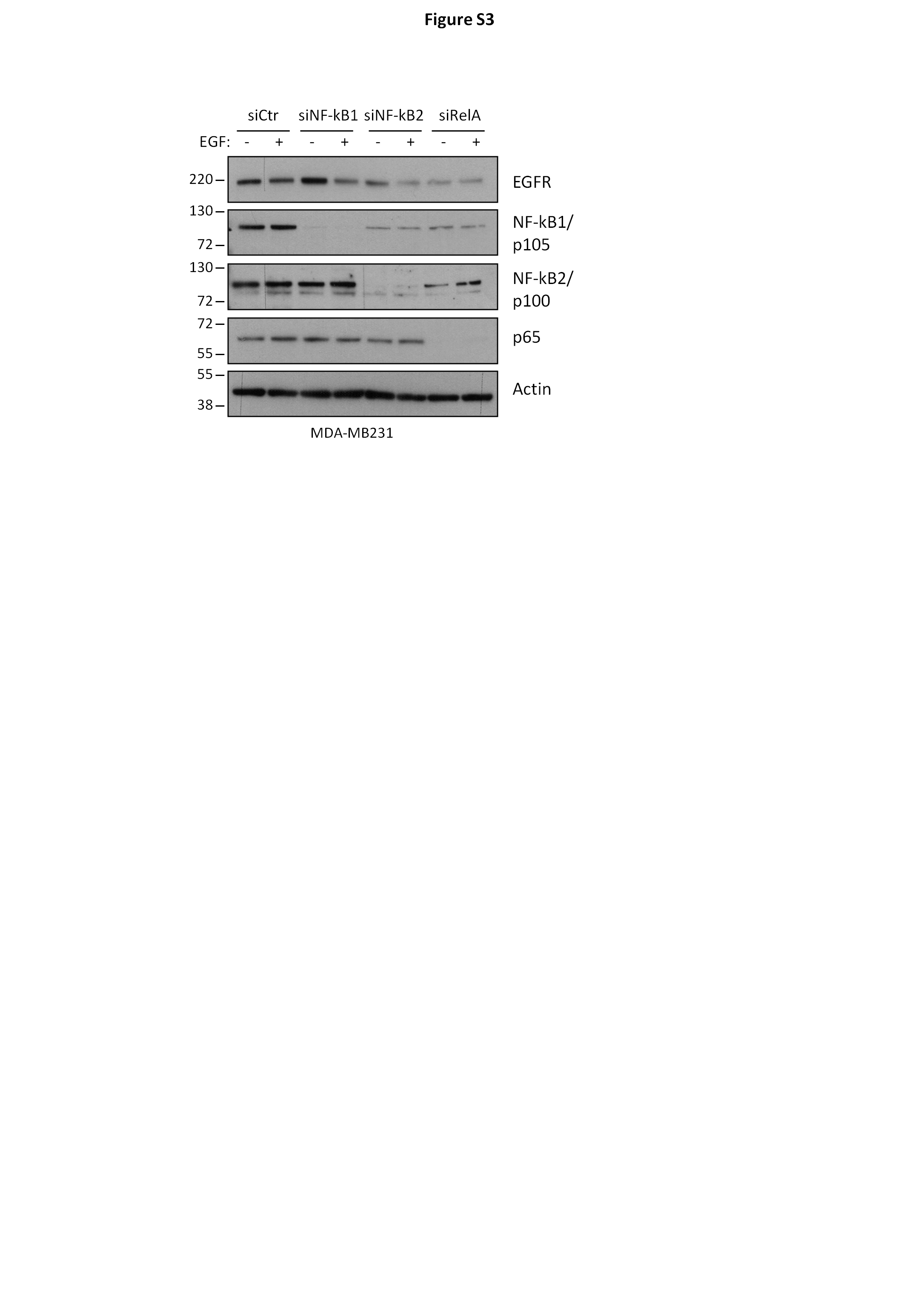

Supplement: Supplementary file 3 — Figure Supplementary S3(TIF 993 kb) [file 41418_2018_100_MOESM3_ESM.tif]

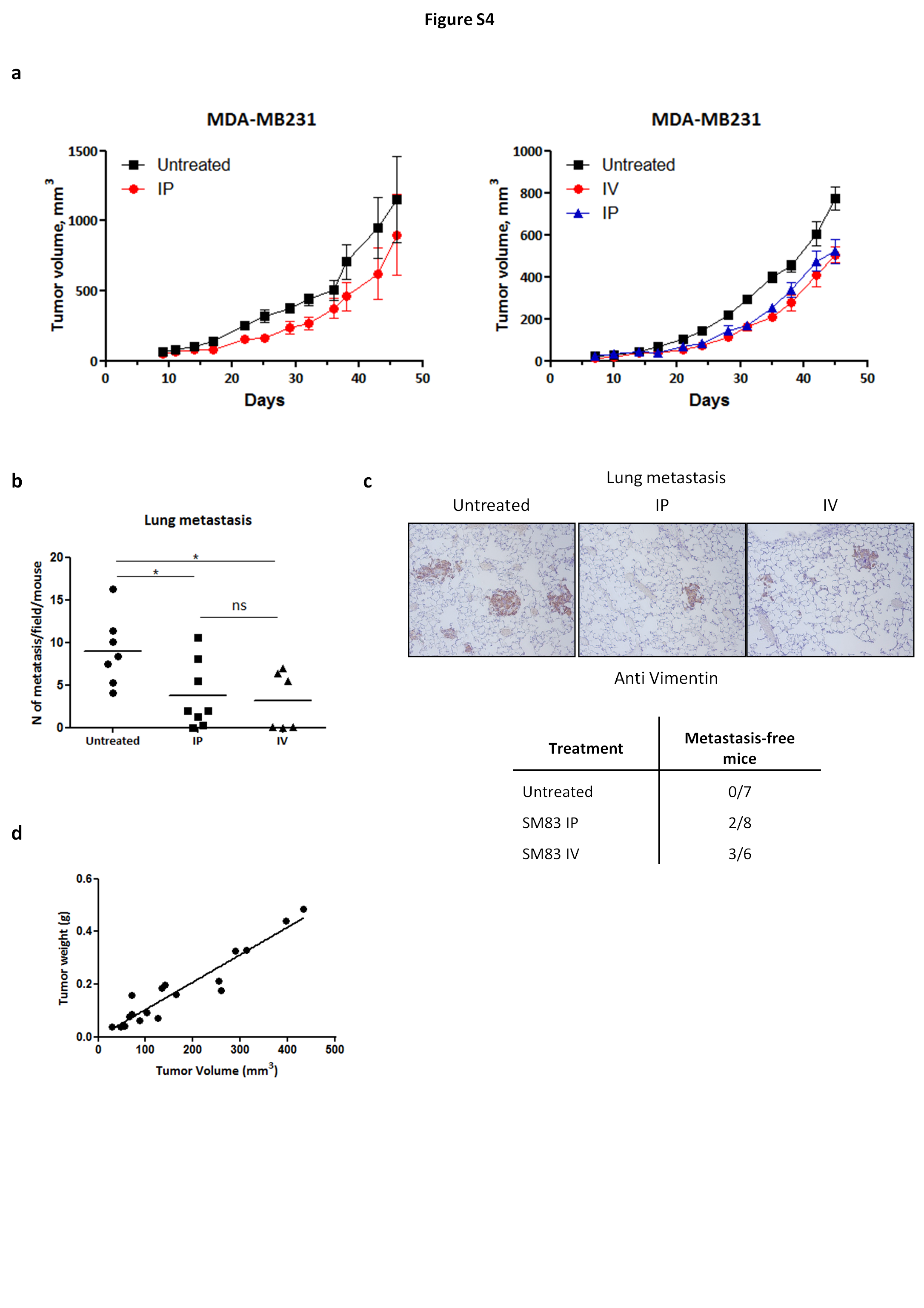

Supplement: Supplementary file 4 — Figure Supplementary S4(TIF 2116 kb) [file 41418_2018_100_MOESM4_ESM.tif]

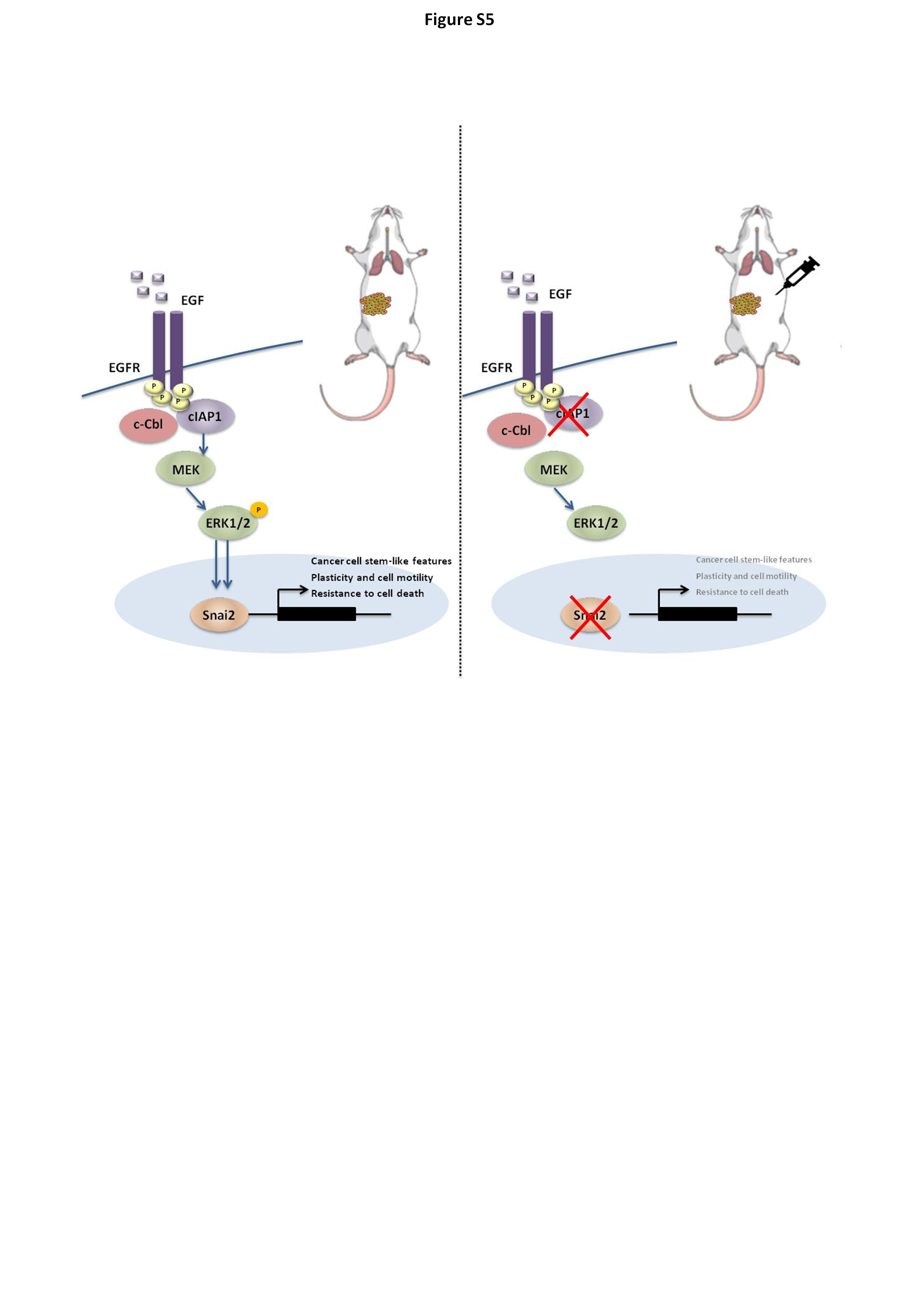

Supplement: Supplementary file 5 — Figure Supplementary S5(TIF 1889 kb) [file 41418_2018_100_MOESM5_ESM.tif]

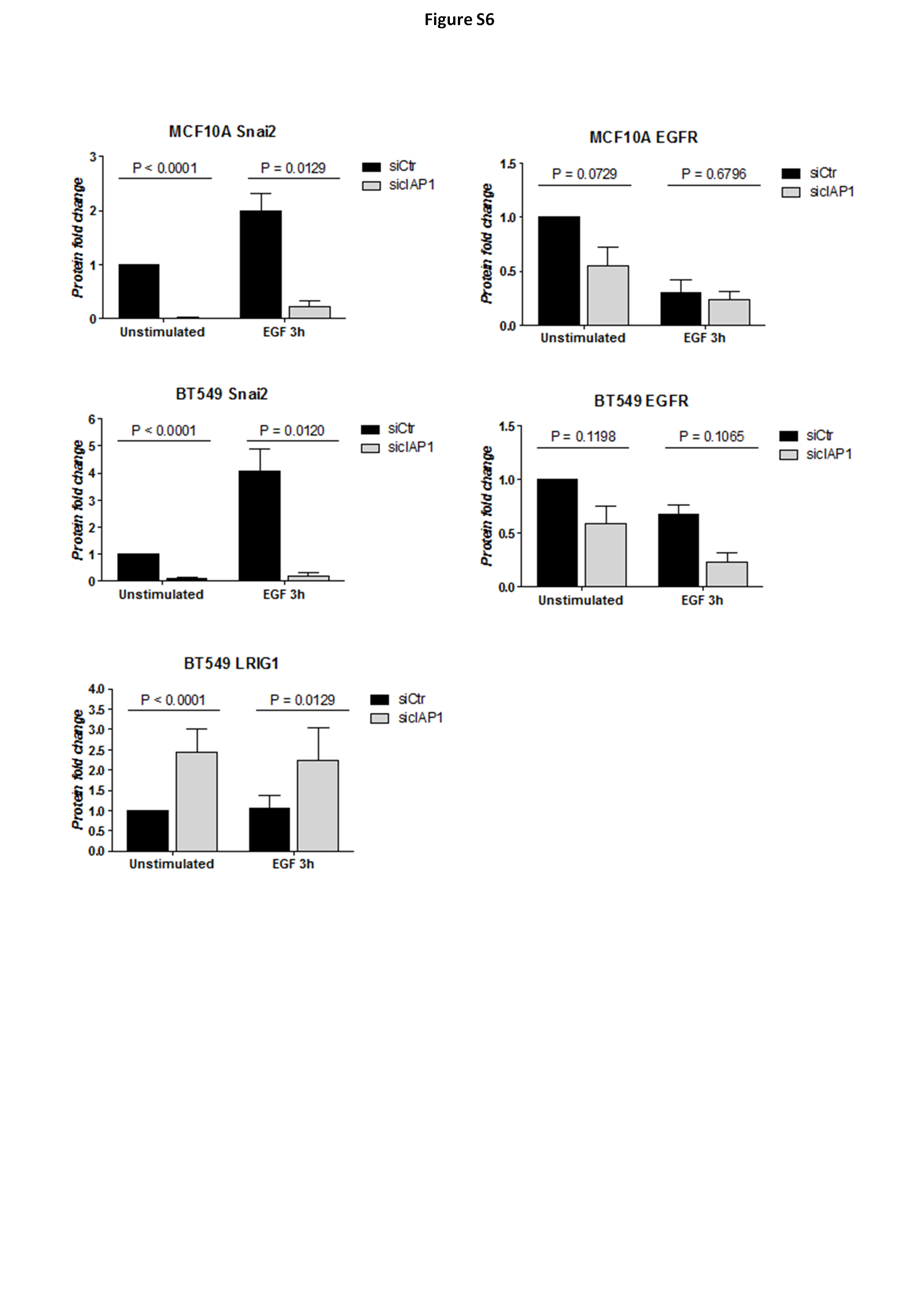

Supplement: Supplementary file 6 — Figure Supplementary S6(TIF 1361 kb) [file 41418_2018_100_MOESM6_ESM.tif]
