# Supplementary material for: cIAP1 regulates the EGFR/Snai2 axis in triple-negative breast cancer cells
Source: Cell Death Differ. 2018 Apr 19;25(12):2147–64. doi: 10.1038/s41418-018-0100-0 (PMC6262016; doi:10.1038/s41418-018-0100-0)
Supplement: Supplementary file 7 — Supplementary figure legends(DOCX 14 kb) [file 41418_2018_100_MOESM7_ESM.docx]

Figure S1. In vivo *treatment with SM83 perturbs the expression of genes involved in TNF and NF-kB signaling pathway*.

Gene Set Enrichment Analysis (GSEA) analysis of the genes significantly up-regulated (50 genes) and down-regulated (15 genes) in MDA-MB231 subcutaneous nodules (Figure 1d) by SM83 treatment confirmed a modification of the expression of genes involved in TNF and NF-kB signaling pathways.

Figure S2. *Silencing of Snai2 reduces the motility properties of MDA-MB231* cells in vitro.

(a) The 15 genes which resulted down-regulated in subcutaneous MDA-MB231 nodules upon treatment with SM83 (Figure 1d) were individually silenced *in vitro* in MDA-MB231 cells. Cell motility was then tested in wound-healing experiments by seeding 4x10^4^ cells in Ibidi chambers, which were cultured overnight and then, after removal of the insert, images were acquired every hour in a Cell-IQ instrument and analyzed with the integrated software. Graphs represent the average of at least 4 independent experiments. (b) MDA-MB231 cells transfected in the same way were also seeded in 96-well plates and viability assessed by CellTiter-Glo assay. List of Dharmacon siRNA pools:

| **Pool Catalog Number** | **Gene Symbol** |
| --- | --- |
| M-003155-02 | MERTK |
| M-005563-02 | GPER |
| M-017626-00 | BDNF |
| M-012633-02 | CTGF |
| M-009328-02 | PTPRU |
| M-016029-01 | OSR1 |
| M-010911-00 | KLHL3 |
| M-009900-01 | RGS4 |
| M-008705-01 | FOXQ1 |
| M-004788-00 | HSDL1 |
| M-025119-01 | RNF144B |
| M-017386-00 | SNAI2 |
| M-005130-02 | PAPPA |
| D-001206-13 | Non-targeting #1, NT1 |

Figure S3. *EGFR expression is regulated by the NF-kB pathway*.

MDA-MB231 cells were transfected with a control siRNA or siRNAs specific for NF-kB1, NF-kB2 and RelA and analyzed by western blot to detect the levels of endogenous EGFR.

Figure S4. *SM83 treatment reduces the growth of primary tumors and displays anti-metastasis activity.*

NOD/SCID mice were engrafted subcutaneously with MDA-MB231 cells and, after two weeks, were treated for 3 weeks with intraperitoneal (IP) and intravenous (IV) injections of SM83 (5 mg/Kg, 5 times/week) in two independent experiments. Mice were killed 2 weeks after the last injection. (a) Tumor volumes and (b) number of lung metastases (Untreated vs IP *P = 0.0238, Untreated vs IV *P = 0.0190, IP vs IV not significant; Unpaired two-tailed t test) detected by (c) anti-Vimentin IHC (upper panel). A table summarizing the metastasis-free MDA-MB231-bearing mice is shown (bottom panel). (d) Primary subcutaneous tumor volumes inferred by caliper measurements were plotted together with the actual tumor weight measured after tumor collection (R^2^ = 0.9011).

Figure S5. *Schematic of the proposed mechanism for SM83 anti-cancer activity.* The targeting of IAPs, and in particular of cIAP1, prevents the activation of the ERK1/2 pathway upon EGFR stimulation. This impairment eventually results in a reduced expression of Snai2, which is an EMT mediator known to be associated with increased cancer aggressiveness, and stem-like and metastasis formation properties.

Figure S6. *Densitometric* a*nalysis of western blots.* Levels of Snai2 (n = 4) and EGFR (n = 4) in MCF10A cells, and Snai2 (n = 6), EGFR (n = 3) and LRIG1 (n = 3) in BT549 cells calculated from n independent experiments.
